# The burden of gastric cancer attributed to high salt intake and predictions through the year 2042: a cross-national comparative analysis of China, Japan, and South Korea

**DOI:** 10.3389/fnut.2025.1584400

**Published:** 2025-07-08

**Authors:** Wei Liu, Zhen-zhen Peng, Dong-qin Zhao, Yang Liu, Kui Liao

**Affiliations:** 1 Department of Oncology, The First Affiliated Hospital of Chongqing Medical University, Chongqing, China; 2 Department of Gastrointestinal Surgery, The First Affiliated Hospital of Chongqing Medical University, Chongqing, China; 3 Chongqing Youth Vocational & Technical College, Chongqing, China

**Keywords:** gastric cancer, high salt intake, Global Burden of Disease, regional comparison, dietary risk factors

## Abstract

**Background:**

Gastric cancer remains a significant health burden, particularly in East Asia, where high salt intake is a major risk factor. This study assesses the gastric cancer burden attributable to high salt intake in China, Japan, and South Korea.

**Methods:**

We analyzed data from the GBD 2021 database, including age-standardized mortality rates (ASMR), age-standardized DALY rates (ASDR), and population attributable fraction (PAF) related to high salt intake. The study focused on individuals aged 25 and above, covering global, Chinese, Japanese, and South Korean populations, with trends from 1990 to 2021 and projections through 2042.

**Results:**

From 1990 to 2021, the gastric cancer burden attributable to high salt intake significantly decreased globally and in China, Japan, and South Korea. Globally, ASMR decreased from 1.74 per 100,000 in 1990 to 0.89 per 100,000 in 2021 (EAPC = −2.26). In China, ASMR decreased from 3.85 per 100,000 in 1990 to 1.78 per 100,000 in 2021 (EAPC = −2.56), with similar declines in Japan and South Korea. Gender disparities remain, with men bearing a significantly higher gastric cancer burden, especially among the elderly.

**Conclusion:**

While high salt intake’s contribution to gastric cancer decreased from 1990 to 2021, it remains a major factor in mortality and DALYs, particularly among elderly and male populations. However, these findings should be interpreted with caution due to reliance on modeled population-level data and the inability to establish causality from observational sources.

## 1 Introduction

Gastric cancer (GC) is a prevalent malignancy that remains a substantial global health burden (1, 2). According to the 2022 global cancer statistics report 968,300 new GC cases worldwide (representing 4.90% of all cancer cases) and 659,800 deaths (representing 6.80% of all cancer-related mortality) (1). The Global Burden of Disease (GBD) Study 2019 show that East Asia accounts for 62.87% of global GC cases, underscoring a marked regional disparity (3). Gastric cancer development is influenced by a combination of environmental and genetic factors, including *Helicobacter pylori* (*H. pylori*) infection, unhealthy lifestyles, and inherited susceptibility. Dietary factors also play a pivotal role in its development (4). In particular, excessive salt intake is not only an independent risk factor for hypertension and cardiovascular diseases (5), but also strongly associated with the onset and progression of GC. A meta-analysis indicates that the risk of GC in populations with high and moderate salt intake increased by 68 and 41%, respectively, compared to those with low intake (6).

Previous studies have demonstrated that excessive salt intake promotes gastric cancer development through several mechanisms. These mechanisms include promoting *Helicobacter pylori* (*H. pylori*) colonization in the gastric mucosa, directly damaging the mucosa, inducing chronic or atrophic gastritis, and accelerating intestinal metaplasia (7, 8). Thus, excessive salt intake is regarded as a major environmental risk factor for GC. Although global salt consumption has declined, countries such as China, Japan, and South Korea, where traditional diets include high-salt foods (such as pickled products, soy sauce, and miso), still report high gastric cancer incidence (9–11).

Although numerous studies have established the relationship between high salt intake and GC (12, 13), most focus on individual countries or regions, lacking cross-national comparative analysis, despite consistent evidence that high salt consumption is a major risk factor. The GBD database offers detailed global disease burden data, but cross-national comparisons on the GC burden attributable to high salt intake are still limited, with few studies comprehensively comparing global data and multiple high-risk countries (e.g., China, Japan, South Korea). Therefore, this study performs a cross-national comparative analysis of high salt intake and gastric cancer burden using GBD data, focusing on countries with high gastric cancer incidence, such as China, Japan, and South Korea. By comparing data across countries, we aim to provide targeted recommendations for public health policies and support the development of more effective gastric cancer prevention in East Asia, where high-salt dietary patterns is widespread.

## 2 Materials and methods

### 2.1 Data source

Data for this study were obtained from the Global Burden of Disease (GBD) 2021 database, developed by the Institute for Health Metrics and Evaluation (IHME). The database includes data from 204 countries and regions, covering multidimensional indicators such as mortality rates, disease burden, quality of life, and risk factors (14, 15). All data were retrieved using the Global Health Data Exchange (GHDx) results tool.^1^

Gastric cancer cases were identified using the International Classification of Diseases, 9th and 10th Revisions (ICD-9 and ICD-10). The specific cancer burden procedures and associated coding information and related codes are available at: http://ghdx.healthdata.org/gbd-2021/code/cod-2. Since GBD 2021 does not provide GC burden attributable to high salt intake data for populations under 25 years, we focus on individuals aged 25 and older, Key indicators included the number of deaths, age-standardized mortality rates (ASMR), disability-adjusted life years (DALYs), and age-standardized DALYs (ASDR). For specific age groups (e.g., 25–29 years, 30–34 years, 35–39 years, and those aged 95 and older), crude rates were used, as GBD 2021 only provides crude rate data for these groups. All data are available through the GBD results tool.^2^

In the GBD 2021 framework, dietary sodium intake was primarily estimated using data from nationally representative 24-h urinary sodium excretion surveys and 24-h dietary recall instruments. Additional sources—including food frequency questionnaires, household expenditure surveys, spot urine samples, and national food sales data—were systematically adjusted and harmonized to approximate 24-h urinary sodium equivalents. High sodium intake was operationally defined as a 24-h urinary sodium excretion exceeding 3 grams per day (g/day), in accordance with established GBD exposure assessment protocols. Notably, these estimates reflect modeled population-level exposures rather than direct individual-level measurements. To support our exposure definitions and modeling approach, we summarized major international thresholds for salt intake, including the WHO guideline, the GBD operational definition, modeled population averages, and risk estimates from prospective meta-analyses (Supplementary Method Table S1) (16–18).

### 2.2 Estimation of high sodium intake-attributed gastric cancer burden

The methods used in the GBD 2021 study are detailed in previous works (14–18). Specifically, spatial–temporal Gaussian process regression (ST-GPR), Cause of Death Ensemble model (CODEm) and disease modeling meta-regression (DisMod-MR) were used to model salt intake levels from 1990 to 2021, estimating exposure levels by age, sex, region, and year. The relative risk (RR) is a key indicator used to assess the impact of different salt intake levels on GC risk. Based on existing literature and meta-regression methods, the RR values for each exposure level were calculated. By combining exposure levels with mortality data, the contribution of high salt intake to the GC burden was evaluated and compared to the theoretical minimum risk exposure level (TMREL). Subsequently, the population attributable fraction (PAF) was calculated using exposure levels, relative risks, and TMREL.

The standard equation for the PAF is as follows (18):

$${\mathrm{PAF}}_{\text{asgt}}\frac{\sum_{x=l}^{u}RR_{\mathrm{asg}}(x)\cdot P_{\text{asgt}}(x)-RR_{\mathrm{asg}}(\text{TMRE}L_{\mathrm{as}})}{\sum_{x=l}^{u}RR_{\mathrm{as}}(x)\cdot P_{\text{asgt}}(x)}$$

where ${\mathrm{PAF}}_{\text{asgt}}$ was the PAF for gastric cancer burden being attributed to high sodium intake for age group a , gender s , location g , and year t . $RR_{\mathrm{asg}}(x)$ was the relative risks between exposure level x (from l to u ) of sodium consumption and gastric cancer separated by age group a , gender s , and year t; $P_{\text{asgt}}(x)$ was the proportion of the population exposed to sodium intake at the level x age group a , gender s , location g , and year t . $\text{TMRE}L_{\mathrm{as}}$ is the TMREL regarding age group a and gender s .

Deaths refer to the number of fatalities occurring within a certain population in the specific period. DALYs (Disability-Adjusted Life Years) is a comprehensive measure that takes into account both the loss of life expectancy and the reduction in quality of life due to disease. The formula for calculating DALYs is: DALYs=YLL+YLD . Here, YLL (Years of Life Lost) represents the years lost due to premature death, while YLD (Years Lived with Disability) measures the years of life lived with disability or reduced quality of life as a result of the disease. This combined approach allows for a more holistic understanding of the disease burden (14, 18).

### 2.3 Statistical analyses

As this study used modeled population-level data from the GBD 2021 database, *a priori* power analysis was not applicable. The GBD framework employs integrated statistical models to ensure sufficient precision in estimates, including built-in uncertainty intervals based on 1,000 draws from the posterior distribution.

Data on deaths, DALYs, ASMR, and ASDR were presented as numbers with 95% UIs on account of the 2.5th and 97.5th percentiles of the above 1,000 estimations (14, 18). The Estimated Annual Percentage Change (EAPC) was used to quantify the trend in the gastric cancer burden attributed to high salt intake from 1990 to 2021. This measure is widely accepted as a reflection of the trend in age-standardized rates (ASR) over time. The ASR can be fitted to a regression model as follows: ln(ASR)=α+βx+ε , where x stands for the calendar year. Then, EAPC could be obtained from the model: EAPC=100×(exp(β)−1) , and its 95% confidence intervals (CIs). If the upper limit of the EAPC and its 95% CI are both negative, the corresponding rate is considered to be decreasing. Conversely, if the lower limit of the EAPC and its 95% CI are both positive, the corresponding rate is considered to be increasing. If the 95% CI includes zero, the corresponding rate is considered to be stable (19, 20).

The Bayesian Age-Period-Cohort (BAPC) prediction model was applied to estimate the GC burden attributed to high salt intake over the next 20 years for global, China, Japan, and South Korea. This model utilized data from 1990 to 2021 as input, considering the effects of age, time, and birth cohort (21). Integrated nested Laplace approximation (INLA) was employed to project trends from 2022 to 2042, allowing for full Bayesian inference. Key features of the BAPC model include: (1) the generation of age-specific and age-standardized predicted rates, and (2) the automatic addition of Poisson noise when interested in the predictive distribution. All statistical analyses were conducted using R software (version 4.2.1). A two-sided *p* value of less than 0.05 was regarded as significant.

## 3 Results

### 3.1 Overview of GC burden attributed to high salt intake in China, Japan, South Korea, and the global

**Figure 1:**
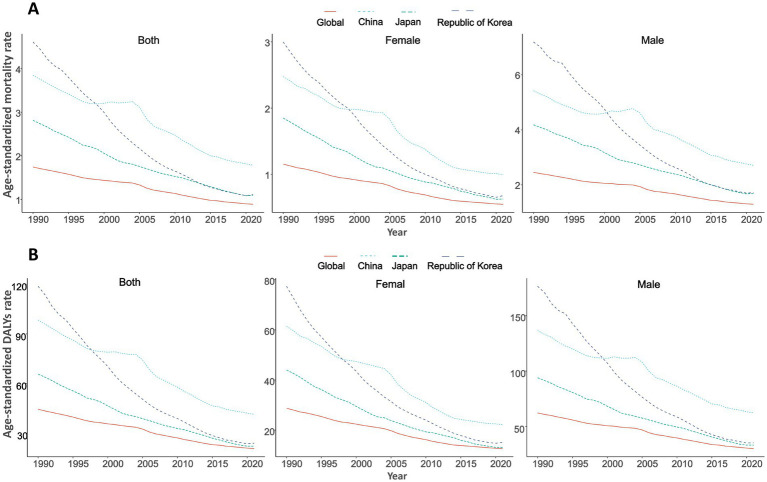
Trends in age-standardized rates of high sodium intake-attributed gastric cancer in China, Japan, South Korea, and globally, 1990–2021, by sex. **(A)** Age-standardized mortality rate; **(B)** Age-standardized DALYs rate.

The GC burden attributed to high salt intake significantly declined from 1990 to 2021 in global China, Japan, South Korea. Globally, the age-standardized mortality rate (ASMR) decreased from 1.74/100,000 in 1990 to 0.89/100,000 in 2021 (EAPC = −2.26) (Table 1; Figure 1A), while age-standardized DALYs (ASDR) dropped from 44.53/100,000 to 20.78/100,000 (EAPC = −2.56) (Table 1; Figure 1B; Supplementary Table S2). The number of deaths slightly increased from 67,844 in 1990 to 75,661 in 2021, while DALYs experienced a modest decline from 1,845,616 to 1,804,591 (Table 2).

**Table 1:** Age-standardized mortality and DALYs rates, and temporal trends of high sodium intake-attributed gastric cancer in China, Japan, South Korea, and globally, 1990–2021, by sex.

Measure	ASMR			ASDR		
	1990	2021	1990–2021	1990	2021	1990–2021
	(95% UI, per 100,000 population)		EAPC (95% CI)	(95% UI, per 100,000 population)		EAPC (95% CI)
Both						
Global	1.74 (−0.00, 8.74)	0.89 (−0.00, 4.37)	−2.26 (−2.35, −2.18)	44.53 (−0.00, 222.31)	20.78 (−0.00, 102.38)	−2.56 (−2.65, −2.47)
China	3.85 (−0.00, 18.79)	1.78 (−0.00, 8.81)	−2.56 (−2.78, −2.34)	98.40 (−0.00, 478.50)	41.46 (−0.00, 208.59)	−2.91 (−3.11, −2.72)
Japan	2.81 (0.00, 13.86)	1.09 (0.00, 5.46)	−3.08 (−3.13, −3.04)	65.73 (0.00, 322.56)	22.26 (0.00, 111.56)	−3.52 (−3.57, −3.47)
South Korea	4.61 (0.00, 22.14)	1.11 (0.00, 5.50)	−4.99 (−5.15, −4.83)	118.73 (0.00, 580.64)	24.08 (0.00, 117.95)	−5.43 (−5.55, −5.30)
Male						
Global	2.46 (−0.00, 12.43)	1.29 (−0.00, 6.34)	−2.12 (−2.22, −2.02)	62.20 (−0.00, 314.71)	29.90 (−0.00, 146.65)	−2.42 (−2.52, −2.33)
China	5.42 (−0.00, 26.71)	2.71 (−0.00, 14.07)	−2.23 (−2.46, −2.00)	136.67 (−0.00, 668.68)	62.16 (−0.00, 323.76)	−2.58 (−2.77, −2.38)
Japan	4.18 (0.00, 20.49)	1.68 (0.00, 8.45)	−2.99 (−3.05, −2.93)	93.97 (0.00, 463.10)	33.08 (0.00, 164.98)	−3.39 (−3.46, −3.33)
South Korea	7.19 (−0.00, 35.74)	1.71 (0.00, 8.52)	−5.04 (−5.19, −4.89)	176.68 (−0.00, 876.88)	35.20 (0.00, 176.51)	−5.53 (−5.66, −5.40)
Female						
Global	1.15 (0.00, 5.86)	0.55 (0.00, 2.79)	−2.57 (−2.67, −2.48)	28.71 (−0.00, 146.18)	12.61 (0.00, 64.62)	−2.84 (−2.94, −2.73)
China	2.48 (−0.00, 12.12)	0.99 (0.00, 5.06)	−3.22 (−3.45, −2.98)	61.39 (−0.00, 302.16)	22.15 (0.00, 112.91)	−3.60 (−3.83, −3.38)
Japan	1.85 (0.00, 9.25)	0.63 (0.00, 3.22)	−3.52 (−3.58, −3.46)	43.97 (0.00, 217.15)	13.04 (0.00, 66.32)	−3.97 (−4.02, −3.92)
South Korea	3.00 (0.00, 14.66)	0.68 (0.00, 3.30)	−5.17 (−5.34, −5.01)	77.39 (0.00, 378.65)	15.09 (0.00, 74.72)	−5.47 (−5.60, −5.34)

ASMR, age-standardized mortality rate; ASDR, age-standardized DALYs rate; DALYs, disability-adjusted life years; UI, uncertainty interval; CI, confidence interval.

**Table 2:** Number of deaths and DALYs cases of high sodium intake-attributed gastric cancer in China, Japan, South Korea, and globally, 1990–2021, by sex.

Measure	Deaths number (95% UI)		DALYs number (95% UI)	
	1990	2021	1990	2021
Both				
Global	67844.54 (−0.00, 339512.73)	75661.15 (−0.00, 372194.01)	1845616.80 (−0.03, 9206157.73)	1804591.52 (−0.00, 8884379.02)
China	31208.21 (−0.00, 152475.63)	36957.67 (−0.00, 183972.30)	892814.81 (−0.04, 4340139.74)	883434.61 (−0.01, 4461210.86)
Japan	4680.05 (0.00, 23010.52)	4801.23 (0.00, 24191.03)	110925.63 (0.00, 544308.90)	76536.72 (0.00, 383663.82)
South Korea	1348.47 (0.00, 6571.07)	1028.66 (0.00, 5083.89)	40195.08 (0.00, 196098.10)	21757.16 (0.00, 106997.82)
Male				
Global	43642.27 (−0.00, 220825.45)	50373.79 (−0.00, 247168.29)	1221058.35 (−0.04, 6151893.29)	1231290.32 (−0.02, 6026424.24)
China	21062.64 (−0.00, 103279.25)	26171.28 (−0.00, 136689.07)	615929.74 (−0.04, 3026941.79)	643007.62 (−0.02, 3367598.78)
Japan	2930.60 (0.00, 14378.56)	3046.48 (0.00, 15263.52)	70972.34 (0.00, 350693.08)	51458.20 (0.00, 257834.32)
South Korea	848.77 (−0.00, 4213.17)	668.64 (0.00, 3358.65)	25588.03 (−0.00, 125362.33)	14752.24 (−0.00, 74327.59)
Female				
Global	24202.27 (0.00, 123377.92)	25287.36 (0.00, 129117.91)	624558.45 (−0.00, 3176862.29)	573301.21 (0.00, 2940974.24)
China	10145.56 (−0.00, 49845.23)	10786.39 (0.00, 55029.75)	276885.07 (−0.00, 1368558.77)	240426.98 (0.00, 1225234.46)
Japan	1749.46 (0.00, 8754.30)	1754.74 (0.00, 9162.57)	39953.29 (0.00, 197553.09)	25078.52 (0.00, 129091.83)
South Korea	499.70 (0.00, 2444.76)	360.02 (0.00, 1757.98)	14607.06 (0.00, 71876.57)	7004.92 (0.00, 34610.02)

DALYs, disability-adjusted life years; UI, uncertainty interval.

In China, the ASMR decreased from 3.85/100,000 in 1990 to 1.78/100,000 in 2021 (EAPC = −2.56), while the ASDR dropped from 98.40/100,000 to 41.46/100,000 (EAPC = −2.91). Data from Japan indicate that the ASMR decreased from 2.81/100,000 in 1990 to 1.09/100,000 in 2021 (EAPC = −3.08), and the ASDR declined from 65.73/100,000 to 22.26/100,000 (EAPC = −3.52). South Korea showed the most significant decrease, with the ASMR falling from 4.61/100,000 in 1990 to 1.11/100,000 in 2021 (EAPC = −4.99), and the ASDR decreasing from 118.73/100,000 to 24.08/100,000 (EAPC = −5.55) (Table 1; Figures 1A,B; Supplementary Figure S1).

### 3.2 Sex disparity of GC burden attributed to high salt intake

Globally, the ASMR for males decreased from 2.46/100,000 in 1990 to 1.29/100,000 in 2021 (EAPC = −2.12%), while the ASDR decreased from 62.20/100,000 to 29.90/100,000 (EAPC = −2.42%). In contrast, the ASMR for females decreased from 1.15/100,000 to 0.55/100,000 (EAPC = −2.57%), and the ASDR declined from 28.71/100,000 to 12.61/100,000 (EAPC = −2.84%) (Table 1; Figure 1). In China, from 1990 to 2021, the ASMR for males decreased from 5.42/100,000 to 2.71/100,000 (EAPC = −2.23%), and the ASDR dropped from 136.67/100,000 to 62.16/100,000 (EAPC = −2.58%). For females, the ASMR decreased from 2.48/100,000 to 0.99/100,000 (EAPC = −3.22%), and ASDR fell from 61.39/100,000 to 22.15/100,000 (EAPC = −3.60%) (Table 1; Figures 1A,B; Supplementary Tables S1, S2). However, the number of deaths for both genders increased, from 21,062 to 26,171 for males and from 10,146 to 10,786 for females (Table 2).

In Japan, from 1990 to 2021, the ASMR for males decreased from 4.18/100,000 to 1.68/100,000 (EAPC = −2.99), and ASDR decreased from 93.97/100,000 to 33.08/100,000 (EAPC = −3.39). For females, the decrease was more significant, with the ASMR falling from 1.85/100,000 to 0.63/100,000 (EAPC = −3.52), and ASDR decreasing from 43.97/100,000 to 13.04/100,000 (EAPC = −3.97) (Tables 1, 2; Figure 1). In South Korea, from 1990 to 2021, the changes were particularly striking: the ASMR for males decreased from 7.19/100,000 to 1.71/100,000 (EAPC = −5.04), and ASDR dropped from 176.68/100,000 to 35.20/100,000 (EAPC = −5.53), with deaths declining from 848 to 669. For females, the decrease was the most significant, the ASMR decreased from 3.00/100,000 to 0.68/100,000 (EAPC = −5.17), and ASDR decreased from 77.39/100,000 to 15.09/100,000 (EAPC = −5.47). The number of deaths for females dropped from 499 to 360 (Tables 1, 2; Figure 1; Supplementary Tables S1, S2).

### 3.3 Age composition of GC burden attributed to high salt intake in 2021

**Figure 2:**
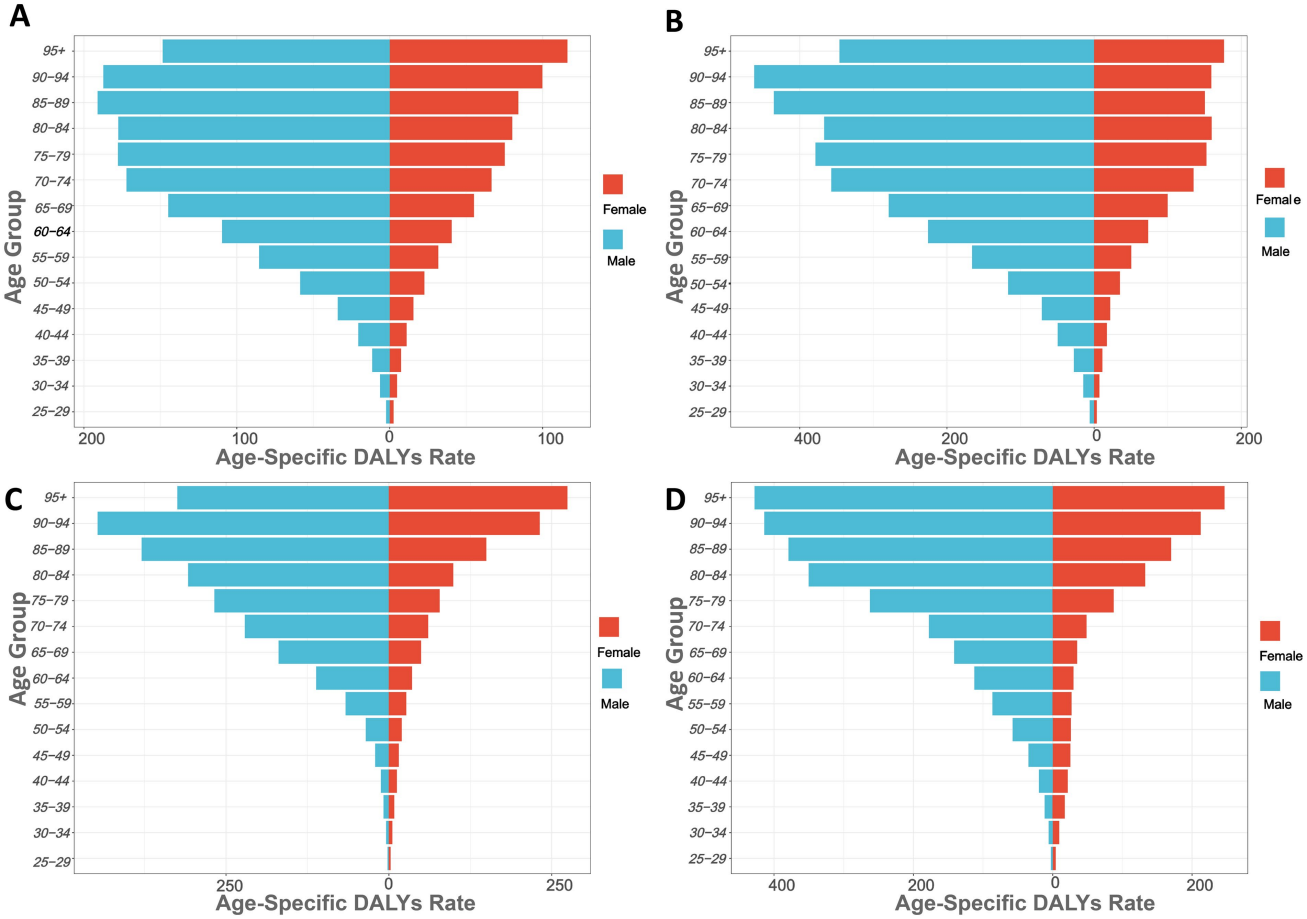
Age-specific DALYs rates of high sodium intake-attributed gastric cancer in 2021, by sex. **(A)** Globally; **(B)** China; **(C)** Japan; **(D)** South Korea.

Figure 2 shows the gastric cancer burden attributed to high salt intake across different age groups globally and in China, Japan, and South Korea (measured in DALYs per 100,000). The results show that the DALYs increases progressively with age. The DALYs of GC burden usually increased sharply after 45 years of age and reached a peak at the age of 85 in the world and the three countries. Globally, the DALYs are highest for males aged 85–89 and females aged 95 and older, at 191.06/100,000 and 116.10/100,000, respectively, indicating the most severe gastric cancer burden in these age groups. In most age groups, the DALYs for males are higher than those for females, particularly in the 75–79 age group, where males have DALYs of 177.70/100,000 and females have 75.19/100,000. Younger age groups, such as 25–29 years, show lower DALYs, with males at 2.36/100,000 and females at 2.44/100,000 (Figure 2A; Supplementary Table S3).

In China, the DALYs for males in the 90–94 age group are the highest at 461.95. The increase in DALYs becomes particularly significant in individuals aged 60 and above. For instance, in the 60–64 age group, the DALYs for males are 225.95, while for females they are 73.36; in the 80–84 age group, the DALYs for males are 367.01, and for females, they are 159.63 (Figure 2B). In Japan (Figure 2C), the DALYs for males in the 90–94 age group are the highest at 447.15. In the 60–64 age group, the DALYs for males are 111.70, while for females they are 35.83; in the 80–84 age group, the DALYs for males are 308.38, and for females, they are 98.98. In South Korea (Figure 2D), the DALYs for males in the 75–79 age group significantly increase, rising from 177.73 in the 70–74 age group to 262.24 in the 75–79 age group. The DALYs for males in the 80–84 age group are 350.39 (Supplementary Tables S4–S6).

### 3.4 Prediction of DALYs for GC burden attributed to high salt intake

**Figure 3:**
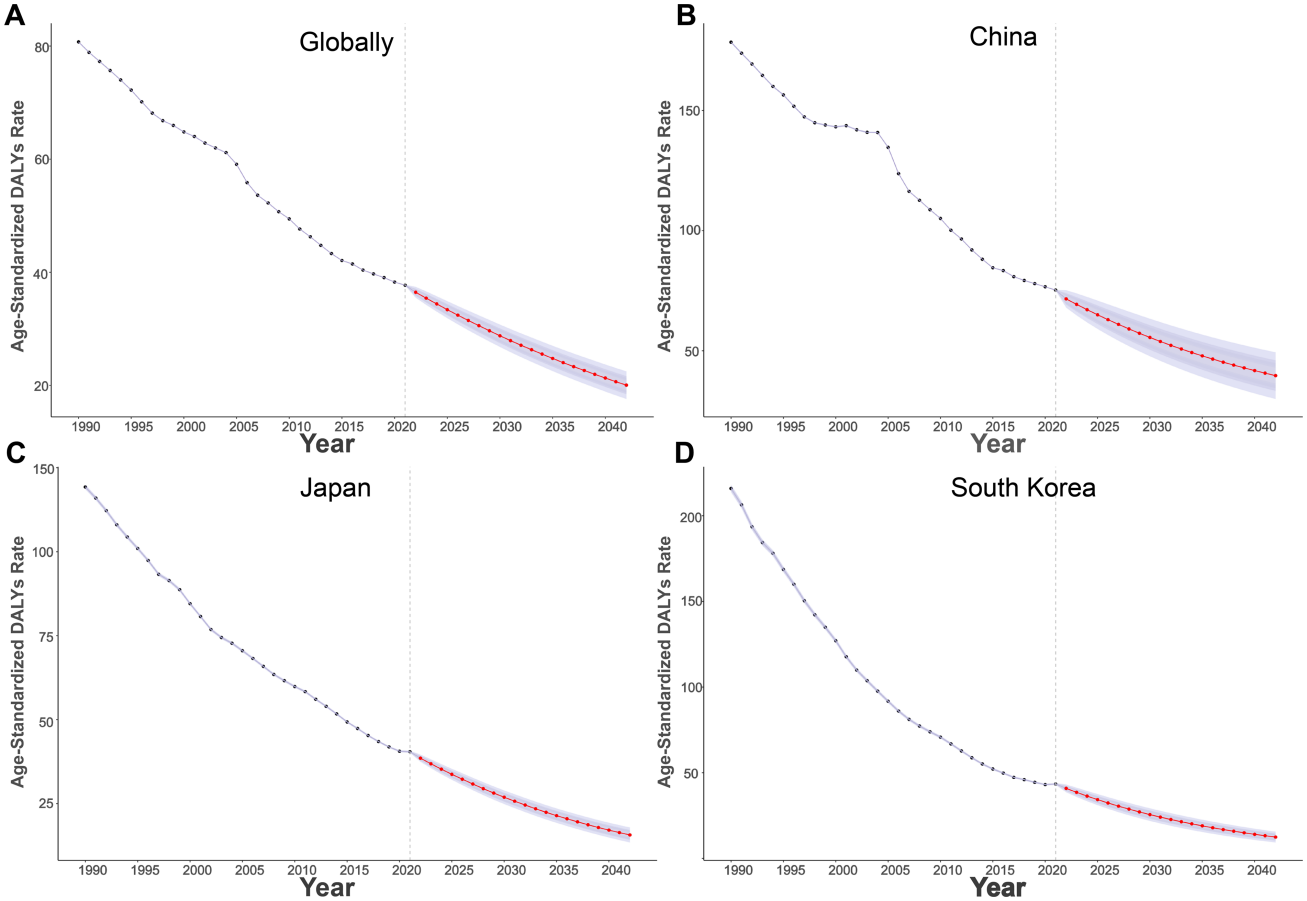
Projections of age-standardized DALYs rates of high sodium intake-attributed gastric cancer from 2022 to 2042. **(A)** Globally; **(B)** China; **(C)** Japan; **(D)** South Korea.

Globally (Figure 3A), the ASDR has been steadily decreasing from 1990 to 2021, and it is projected to further decrease to around 20/100,000 by 2042. In China (Figure 3B), the ASDR has also decreased, with an expected reduction to nearly 35/100,000 by 2042. Similarly, Japan (Figure 3C) and South Korea (Figure 3D) show a downward trend, with Japan’s ASDR projected to reach approximately 12.5/100,000, and South Korea’s to approach 15/100,000 by 2042.

## 4 Discussion

This study evaluates the impact of high salt intake on the gastric cancer (GC) burden in China, comparing it with global data and data from Japan and South Korea, based on the GBD 2021 database. We used a combination of indicators, including ASMR, ASDR, and PAF, to quantify the contribution of high salt intake to the gastric cancer burden. The results indicate that high salt intake significantly impacts gastric cancer mortality and DALYs, remaining a major contributor to the GC burden.

Although global salt intake has decreased, traditional dietary habits (e.g., consuming pickled foods, soy sauce, and miso) remain prevalent in the studied regions, contributing to significantly higher GC rates compared to the global average (3). A dietary survey in South Korea found that foods (e.g., ramen, baechukimchi, and doenjang-guk) significantly contributed to sodium intake, with consumption 1.3 to 2.3 times higher than the recommended levels (9). Japan’s dietary habits still predominantly feature high salt, particularly due to the widespread use of traditional seasonings like soy sauce, miso, and dried fish, contributing to high salt intake (11, 12). Although Japan has set salt intake targets (less than 7.5 g/day for males and less than 6.5 g/day for females), these levels remain above the international recommendation of 5 g/day (22). A nationwide survey of the Japanese population revealed a significant difference in salt intake between gastric cancer patients and non-patients (7.8 g/day vs. 6.4 g/day, *p* = 0.002). Non-gastric cancer patients consumed 6.3 g/day for males and 6.2 g/day for females, while gastric cancer patients consumed 7.6 g/day for males and 7.1 g/day for females (23). To enhance transparency and comparability, Supplementary Table S7 summarises the recommended salt intake limits, operational definitions of high-salt exposure (via dietary and urinary sodium), and the associated relative risks (RR) of gastric cancer. These thresholds support the epidemiological foundation of our modeling approach and provide context for interpreting burden differences across countries.

Since 2007, China has introduced several salt reduction measures, including changes to nutrition labeling standards for pre-packaged foods and the promotion of nutrition and health education (24). The “China Nutrition and Chronic Disease Status Report (2015)” indicates a declining trend in salt intake among the population. For instance, the average daily salt consumption for cooking in 2012 was 10.5 grams, a reduction of 1.5 grams from 2002. This change has helped reduce the GC burden to some extent, but the level remains significantly higher than the World Health Organization’s recommended limit of 5 g/day (7).

China continues to carry a higher gastric-cancer (GC) burden attributable to high salt intake than Japan and South Korea. Several country-specific factors may explain this disparity. In northern China, frequent consumption of pickled vegetables and salted fish—foods typically rich in nitrites—remains common, whereas Japanese and Korean diets include larger amounts of potentially protective items such as fresh produce, seaweed and citrus fruits, which may mitigate salt-related risk (10, 11, 25). In addition, variability in salt quality—particularly the occasional use of less-refined, impurity-rich salt—and higher groundwater nitrate levels reported for some Chinese provinces could further elevate GC risk (26, 27). Public awareness of recommended sodium limits is also lower in China; only 6.1% of adults correctly identify the daily guideline (28).

Differences in policy timing, enforcement and healthcare infrastructure contribute to the divergent downward trends. Japan introduced national sodium targets (< 8 g /day) as early as the 1980s and has maintained mandatory front-of-pack labelling and regular diet surveys (29). South Korea’s 2012 National Plan to Reduce Sodium Intake spurred large-scale reformulation of kimchi, instant noodles and school meals, leading to a 23% reduction in population sodium intake between 2010 and 2021 (30). By contrast, China’s salt-reduction agenda (Healthy China 2030) was launched more recently, combining high-sodium warning labels, community education and regional trials of potassium-salt substitutes (31).

This study also found that the GC burden is significantly higher in the elderly population than in younger groups. This is closely related to higher sodium intake, decreased gastrointestinal function, and a more limited diet in older adults. For example, individuals in the 60–64, 80–84, and 85 + age groups have higher sodium intake compared to other age groups. This may be associated with the decline in digestive and chewing functions in older adults, which in turn leads to a reduction in dietary diversity (32).

In this study, we found that the age-specific mortality rate and DALY rate for GC due to high salt intake are significantly higher in males than in females, which is consistent with previous research (22). This disparity may be attributed to dietary habits and lifestyle factors in the male population, such as higher salt intake and unhealthy behaviors like smoking and alcohol consumption (33–35). Studies have shown that males excrete more salt through urine (247 mmol/d) compared to females (218 mmol/d), suggesting that males are more likely to be exposed to higher internal sodium levels, which in turn leads to a more severe disease burden. Evidence suggests that women who smoke or consume alcohol may experience elevated GC risk under high-salt dietary conditions, though generally to a lesser extent than men. A large cohort study from Korea reported that the synergistic effect of smoking and high sodium intake increased GC risk in both sexes, but the magnitude was higher in males than females (36). Similarly, Japanese studies found that female smokers with high salt consumption had a modestly increased risk of non-cardia gastric cancer, though statistical significance was often limited by smaller female smoker sample sizes (23). These findings support the hypothesis that behavioral exposures—rather than sex alone—may partially explain the observed disparities in GC burden.

Reducing sodium intake remains a primary nutritional goal for many countries. Numerous high-income countries have implemented proactive measures. For example, the United States regulates the salt content in processed and fast foods to promote reduced intake. Singapore focuses on promoting fruit and vegetable consumption through education to reduce salt use. Canada requires mandatory sodium labeling on packaged foods to support informed consumer choices and facilitate sodium reduction Finland recommends using a salt substitute called “Pansalt^®^” to reduce sodium levels in food (37). Collectively, these country-level strategies offer valuable insights for shaping global sodium reduction initiatives.

Predictive analysis shows that the GC burden from high salt intake has been decreasing annually in global, Chinese, Japanese, and South Korean populations. Moving forward, strengthening international experience-sharing and collaboration is crucial to promoting global efforts to reduce high salt intake (38, 39). Through salt reduction policies, improved dietary habits, and nutrition education, the contribution of high salt diets to the gastric cancer burden can be significantly reduced by effective interventions (40).

High salt intake increases the risk of GC through several mechanisms. *In vitro* studies in rats have shown that excessive salt consumption can lead to gastritis and enhance the carcinogenic effects of certain substances, such as N-methyl-N-nitro-N-nitrosoguanidine (41, 42). One key mechanism is that high salt diets promote the colonization and expansion of *Helicobacter pylori*, particularly CagA-positive strains, leading to chronic inflammation, gastric mucosal damage, and the onset of atrophy and intestinal metaplasia, creating a favorable environment for gastric cancer (43). In an endoscopy-based study, Song et al. found that high salt intake increases the risk of gastric atrophy with intestinal metaplasia (44). Moreover, high salt diets are closely linked to increased oxidative stress, which can damage cellular DNA and further drive carcinogenesis (45). High salt intake may also influence gastric cancer development by altering the composition of the gut microbiota. Research indicates that long-term consumption of high-salt foods can disrupt the gastrointestinal microbiome balance, promoting the growth of carcinogenic bacteria and increasing the risk of GC (46).

This study uses large-scale, multidimensional data from the GBD 2021 database, including information on high salt intake and its contribution to gastric cancer burden in global, Chinese, Japanese, and South Korean populations, ensuring broad and representative results. Based on longitudinal data from 1990 to 2021, this study analyzes trends in the GC burden attributable to high salt intake and evaluates differences across gender, age groups, and regions, enhancing the comparability and scientific rigor of the findings. Using comprehensive indicators such as ASMR and ASDR, the study provides an in-depth examination of the impact of high salt intake on gastric cancer.

There are several limitations in this study. First, the GBD 2021 database does not provide gastric cancer burden estimates for individuals under 25 years of age; therefore, our analysis was restricted to populations aged 25 and older, which may underestimate the potential burden among younger individuals. Second, dietary patterns, data quality, and sodium exposure sources vary significantly across countries and regions, which may introduce biases and limit comparability. Third, the study relied on modeled population-level estimates from the GBD framework rather than individual-level data. Specifically, the exposure data for high sodium intake were estimated using a combination of 24-h urinary sodium excretion surveys and adjusted ancillary sources such as dietary recalls, spot urine samples, and food sales data. These sources were harmonized using statistical modeling methods (e.g., ST-GPR, CODEm, DisMod-MR), which may lead to uncertainty in exposure classification.

Additionally, although this study estimated the burden of gastric cancer attributable to high salt intake using GBD 2021 data, it is important to acknowledge that these estimates reflect modeled associations based on prior observational studies rather than direct experimental evidence. As such, causality cannot be definitively established. Observational data are inherently susceptible to residual confounding, reverse causation, and unmeasured socioeconomic or dietary variables. While previous research has identified consistent associations between high salt intake and gastric cancer risk (47, 48), the evidence does not fully satisfy the Bradford Hill criteria for causation. Future studies are warranted to investigate causal mechanisms, ideally integrating individual-level cohort data, biological markers, and intervention outcomes.

## 5 Conclusion

This study assessed the gastric cancer burden attributable to high salt intake in China, Japan, and South Korea using GBD 2021 data. While a declining trend in age-standardized mortality and DALY rates was observed from 1990 to 2021, the burden remains substantial—particularly in China and among elderly males. These findings highlight the continued importance of salt reduction strategies in East Asia. However, as the study relies on modeled observational data rather than individual-level cohorts, the results should be interpreted with caution. Causality cannot be firmly established, and residual confounding factors may exist. Future research should aim to integrate cohort-based evidence and biological mechanisms to strengthen causal inference. Expanding public awareness, enhancing dietary policies, and tailoring salt reduction interventions to regional characteristics remain essential for gastric cancer prevention and control.

## Data Availability

Publicly available datasets were analyzed in this study. This data can be found here: all data used in this study are publicly available and can be accessed from the Global Health Data Exchange (GBD 2021) website, provided by the Institute for Health Metrics and Evaluation (https://ghdx.healthdata.org/gbd-2021).

## Footnotes

^1^ http://ghdx.healthdata.org/gbd-results-tool

^2^ https://www.healthdata.org/gbd/2021

## Glossary

GC: Gastric Cancer
GBD: Global Burden of Disease
YLL: Years of Life Lost
YLD: Years Lived with Disability
DALY: Disability-Adjusted Life Years
ASMR: Age-Standardized Mortality Rate
ASR: Age-standardized rates
ASDR: Age-Standardized DALYs Rate
PAF: Population Attributable Fraction
EAPC: Estimated Annual Percentage Change
*H. pylori*: *Helicobacter pylori*
ST-GPR: Spatial–Temporal Gaussian Process Regression
DisMod-MR: Disease Modeling Meta-Regression
CODEm: Cause of Death Ensemble model
GHDx: Global Health Data Exchange
IHME: Institute for Health Metrics and Evaluation
RR: Relative Risk
TMREL: Theoretical Minimum Risk Exposure Level
CI: confidence interval
UI: Uncertainty Interval
BAPC: Bayesian Age-Period-Cohort
ICD-9/10: International Classification of Diseases, 9th/10th Revision
