# Correction: The burden of gastric cancer attributed to high salt intake and predictions through the year 2042: a cross-national comparative analysis of China, Japan, and South Korea

**DOI:** 10.3389/fnut.2025.1729155

**Published:** 2025-11-17

**Authors:** Wei Liu, Zhen-zhen Peng, Dong-qin Zhao, Yang Liu, Kui Liao

**Affiliations:** 1 Department of Oncology, The First Affiliated Hospital of Chongqing Medical University, Chongqing, China; 2 Department of Gastrointestinal Surgery, The First Affiliated Hospital of Chongqing Medical University, Chongqing, China; 3 Chongqing Youth Vocational & Technical College, Chongqing, China

**Keywords:** gastric cancer, high salt intake, Global Burden of Disease, regional comparison, dietary risk factors

Author Wei Liu was erroneously assigned as corresponding author. The correct corresponding author is Kui Liao (568396796@qq.com).

Wei Liu remains as a co-first author of the article.

The original version of this article has been updated.

