# Supplementary material for: The burden of gastric cancer attributed to high salt intake and predictions through the year 2042: a cross-national comparative analysis of China, Japan, and South Korea
Source: Front Nutr. 2025 Jul 8;12:1584400. doi: 10.3389/fnut.2025.1584400 (PMC12279510; doi:10.3389/fnut.2025.1584400)
Supplement: Supplementary file 1 [file Table_1.DOC]

| 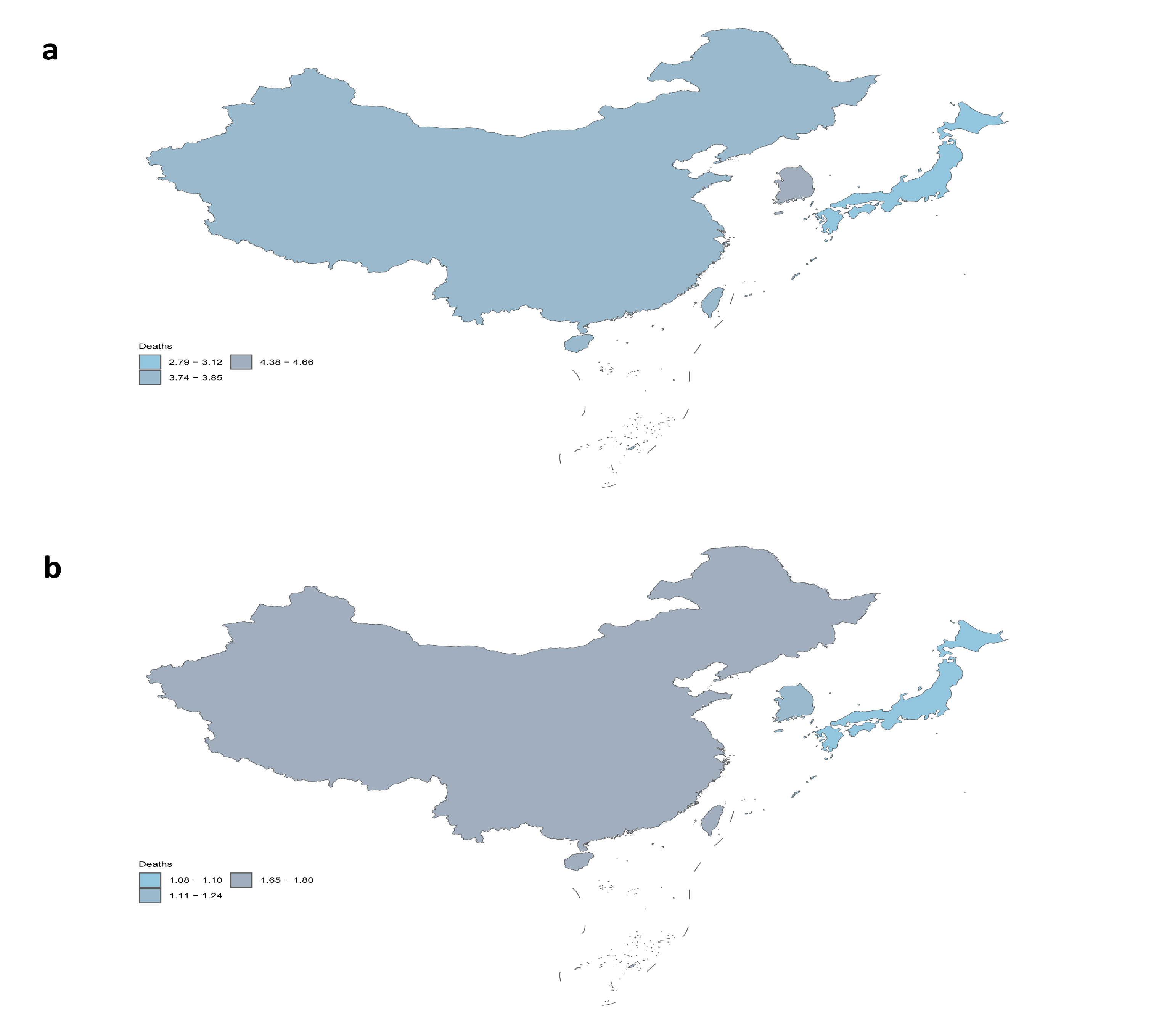 |
| --- |

Supplementary Figure S1. Age-Standardized Mortality Rates of High Sodium Intake-Attributed Gastric Cancer in China, Japan, and South Korea. (a) In 1990; (b) In 2021.

Supplementary Method Table S1.Key sodium-/salt-related thresholds, recommended limits, and their reported associations with gastric cancer (GC)

| Category / IndicatorThreshold or Mean ExposureMeasurement BasisReported GC risk (vs. lowest-exposure group)Principal sourceWHO guideline (adults)< 2 g sodium /day (≈ < 5 g salt)Dietary intake– (not a risk estimate) WHO 2024 factsheetGBD “high sodium” definition≥ 3 g sodium /day 24-h urinary excretion^†^– (definition only)GBD 2021 risk profileGlobal population mean10.8 g salt/ day (2019)Modeled intake (multiple sources)–  WHO salt-reduction report 2023Meta-analysis (prospective cohorts)“High” vs. “low” intake ( ≥ 10 g vs. ≤ 5 g salt/ day)*Dietary intake (FFQ/24-h recall)OR 1.55 (95 % CI 1.45–1.64) Ge & colleagues 2022 |  |  |  | |  |
| --- | --- | --- | --- | --- | --- |
|  |  |  | |  |  |
|  |  |  | |  |  |
|  |  |  | |  |  |
|  |  |  | |  |  |

^†^24-hour urinary sodium is regarded as the gold-standard biomarker for population exposure; dietary instruments are adjusted to its equivalent in the GBD framework.* Cut-points differed slightly across cohorts; most classified the top vs. bottom intake tertiles or quartiles, which generally map to ≥ 10 g vs. ≤ 5 g salt/ day.

Supplementary Table S1. Age-standardized mortality rate of high sodium intake-attributed gastric cancer in China, Japan, South Korea, and the Globally from 1990 to 2021

| location_name | sex_name | year | val | upper | lower |
| --- | --- | --- | --- | --- | --- |
| Global | Male | 1990 | 2.46 | 12.43 | -3.60E-08 |
| Global | Female | 1990 | 1.15 | 5.86 | 3.86E-09 |
| Global | Both | 1990 | 1.74 | 8.74 | -1.53E-08 |
| Global | Male | 1991 | 2.41 | 12.22 | -4.06E-08 |
| Global | Female | 1991 | 1.13 | 5.73 | 5.31E-09 |
| Global | Both | 1991 | 1.71 | 8.62 | -1.36E-08 |
| Global | Male | 1992 | 2.37 | 11.85 | -4.45E-08 |
| Global | Female | 1992 | 1.10 | 5.52 | 4.44E-09 |
| Global | Both | 1992 | 1.67 | 8.38 | -1.80E-08 |
| Global | Male | 1993 | 2.32 | 11.61 | -3.57E-08 |
| Global | Female | 1993 | 1.08 | 5.47 | 3.31E-09 |
| Global | Both | 1993 | 1.64 | 8.20 | -1.50E-08 |
| Global | Male | 1994 | 2.28 | 11.48 | -3.16E-08 |
| Global | Female | 1994 | 1.06 | 5.39 | 5.27E-09 |
| Global | Both | 1994 | 1.61 | 8.12 | -1.26E-08 |
| Global | Male | 1995 | 2.23 | 11.21 | -2.52E-08 |
| Global | Female | 1995 | 1.03 | 5.21 | 6.61E-09 |
| Global | Both | 1995 | 1.58 | 7.89 | -1.11E-08 |
| Global | Male | 1996 | 2.18 | 10.88 | -1.87E-08 |
| Global | Female | 1996 | 1.00 | 5.09 | 6.18E-09 |
| Global | Both | 1996 | 1.54 | 7.69 | -7.85E-09 |
| Global | Male | 1997 | 2.13 | 10.74 | -1.91E-08 |
| Global | Female | 1997 | 0.97 | 4.89 | 8.83E-09 |
| Global | Both | 1997 | 1.50 | 7.47 | -5.57E-09 |
| Global | Male | 1998 | 2.10 | 10.41 | -1.86E-08 |
| Global | Female | 1998 | 0.95 | 4.79 | 1.08E-08 |
| Global | Both | 1998 | 1.47 | 7.30 | 2.00E-11 |
| Global | Male | 1999 | 2.08 | 10.47 | -1.69E-08 |
| Global | Female | 1999 | 0.94 | 4.69 | 8.47E-09 |
| Global | Both | 1999 | 1.46 | 7.31 | -5.70E-10 |
| Global | Male | 2000 | 2.05 | 10.26 | -1.96E-08 |
| Global | Female | 2000 | 0.92 | 4.74 | 7.25E-09 |
| Global | Both | 2000 | 1.44 | 7.13 | -4.04E-09 |
| Global | Male | 2001 | 2.05 | 10.20 | -2.31E-08 |
| Global | Female | 2001 | 0.90 | 4.53 | 6.06E-09 |
| Global | Both | 2001 | 1.42 | 7.10 | -2.84E-09 |
| Global | Male | 2002 | 2.02 | 9.91 | -2.55E-08 |
| Global | Female | 2002 | 0.89 | 4.51 | 7.79E-09 |
| Global | Both | 2002 | 1.40 | 6.94 | -3.19E-09 |
| Global | Male | 2003 | 2.01 | 10.02 | -2.23E-08 |
| Global | Female | 2003 | 0.87 | 4.46 | 7.34E-09 |
| Global | Both | 2003 | 1.39 | 6.99 | -3.03E-09 |
| Global | Male | 2004 | 1.99 | 9.86 | -1.55E-08 |
| Global | Female | 2004 | 0.86 | 4.40 | 7.80E-09 |
| Global | Both | 2004 | 1.38 | 6.87 | -1.45E-09 |
| Global | Male | 2005 | 1.94 | 9.64 | -1.90E-08 |
| Global | Female | 2005 | 0.83 | 4.16 | 9.10E-09 |
| Global | Both | 2005 | 1.33 | 6.58 | -3.40E-10 |
| Global | Male | 2006 | 1.83 | 9.07 | -1.55E-08 |
| Global | Female | 2006 | 0.78 | 3.94 | 6.00E-09 |
| Global | Both | 2006 | 1.26 | 6.27 | 1.25E-09 |
| Global | Male | 2007 | 1.76 | 8.91 | -1.69E-08 |
| Global | Female | 2007 | 0.75 | 3.80 | 6.74E-09 |
| Global | Both | 2007 | 1.21 | 5.98 | 1.78E-09 |
| Global | Male | 2008 | 1.74 | 8.65 | -1.25E-08 |
| Global | Female | 2008 | 0.73 | 3.70 | 5.93E-09 |
| Global | Both | 2008 | 1.19 | 5.95 | 1.98E-09 |
| Global | Male | 2009 | 1.70 | 8.49 | -1.21E-08 |
| Global | Female | 2009 | 0.71 | 3.61 | 3.95E-09 |
| Global | Both | 2009 | 1.16 | 5.81 | -6.50E-10 |
| Global | Male | 2010 | 1.67 | 8.37 | -1.59E-08 |
| Global | Female | 2010 | 0.69 | 3.55 | 3.84E-09 |
| Global | Both | 2010 | 1.14 | 5.69 | -2.56E-09 |
| Global | Male | 2011 | 1.62 | 7.93 | -1.71E-08 |
| Global | Female | 2011 | 0.66 | 3.36 | 4.13E-09 |
| Global | Both | 2011 | 1.10 | 5.47 | -2.62E-09 |
| Global | Male | 2012 | 1.57 | 7.80 | -1.22E-08 |
| Global | Female | 2012 | 0.64 | 3.26 | 3.49E-09 |
| Global | Both | 2012 | 1.07 | 5.35 | -5.16E-09 |
| Global | Male | 2013 | 1.53 | 7.56 | -8.98E-09 |
| Global | Female | 2013 | 0.62 | 3.19 | 2.45E-09 |
| Global | Both | 2013 | 1.04 | 5.16 | -2.42E-09 |
| Global | Male | 2014 | 1.48 | 7.44 | -1.02E-08 |
| Global | Female | 2014 | 0.60 | 3.10 | 2.31E-09 |
| Global | Both | 2014 | 1.01 | 5.07 | -3.21E-09 |
| Global | Male | 2015 | 1.44 | 7.09 | -1.33E-08 |
| Global | Female | 2015 | 0.59 | 3.07 | 2.49E-09 |
| Global | Both | 2015 | 0.98 | 4.87 | -4.35E-09 |
| Global | Male | 2016 | 1.42 | 7.09 | -1.31E-08 |
| Global | Female | 2016 | 0.59 | 3.05 | 3.50E-09 |
| Global | Both | 2016 | 0.97 | 4.80 | -4.35E-09 |
| Global | Male | 2017 | 1.38 | 6.91 | -1.04E-08 |
| Global | Female | 2017 | 0.58 | 3.00 | 2.96E-09 |
| Global | Both | 2017 | 0.94 | 4.76 | -2.91E-09 |
| Global | Male | 2018 | 1.35 | 6.87 | -1.16E-08 |
| Global | Female | 2018 | 0.57 | 2.91 | 3.82E-09 |
| Global | Both | 2018 | 0.93 | 4.66 | -3.84E-09 |
| Global | Male | 2019 | 1.33 | 6.76 | -8.86E-09 |
| Global | Female | 2019 | 0.56 | 2.88 | 3.11E-09 |
| Global | Both | 2019 | 0.91 | 4.65 | -2.64E-09 |
| Global | Male | 2020 | 1.31 | 6.41 | -9.99E-09 |
| Global | Female | 2020 | 0.55 | 2.83 | 2.96E-09 |
| Global | Both | 2020 | 0.90 | 4.45 | -2.71E-09 |
| Global | Male | 2021 | 1.29 | 6.34 | -8.08E-09 |
| Global | Female | 2021 | 0.55 | 2.79 | 3.67E-09 |
| Global | Both | 2021 | 0.89 | 4.37 | -3.71E-09 |
| Japan | Male | 1990 | 4.18 | 20.49 | 1.17E-07 |
| Japan | Female | 1990 | 1.85 | 9.25 | 5.54E-08 |
| Japan | Both | 1990 | 2.81 | 13.86 | 8.67E-08 |
| Japan | Male | 1991 | 4.09 | 19.98 | 1.03E-07 |
| Japan | Female | 1991 | 1.79 | 8.89 | 5.95E-08 |
| Japan | Both | 1991 | 2.74 | 13.44 | 8.11E-08 |
| Japan | Male | 1992 | 3.99 | 19.58 | 9.19E-08 |
| Japan | Female | 1992 | 1.73 | 8.61 | 5.80E-08 |
| Japan | Both | 1992 | 2.66 | 13.12 | 7.80E-08 |
| Japan | Male | 1993 | 3.86 | 18.90 | 9.02E-08 |
| Japan | Female | 1993 | 1.66 | 8.28 | 5.52E-08 |
| Japan | Both | 1993 | 2.57 | 12.66 | 6.96E-08 |
| Japan | Male | 1994 | 3.78 | 18.40 | 8.70E-08 |
| Japan | Female | 1994 | 1.60 | 7.95 | 4.69E-08 |
| Japan | Both | 1994 | 2.50 | 12.30 | 6.67E-08 |
| Japan | Male | 1995 | 3.68 | 18.00 | 8.18E-08 |
| Japan | Female | 1995 | 1.55 | 7.68 | 4.08E-08 |
| Japan | Both | 1995 | 2.43 | 11.97 | 6.82E-08 |
| Japan | Male | 1996 | 3.57 | 17.44 | 8.41E-08 |
| Japan | Female | 1996 | 1.48 | 7.37 | 3.58E-08 |
| Japan | Both | 1996 | 2.34 | 11.56 | 6.52E-08 |
| Japan | Male | 1997 | 3.44 | 16.79 | 1.01E-07 |
| Japan | Female | 1997 | 1.41 | 6.98 | 3.26E-08 |
| Japan | Both | 1997 | 2.24 | 11.05 | 5.98E-08 |
| Japan | Male | 1998 | 3.40 | 16.63 | 9.79E-08 |
| Japan | Female | 1998 | 1.38 | 6.85 | 2.96E-08 |
| Japan | Both | 1998 | 2.21 | 10.92 | 6.13E-08 |
| Japan | Male | 1999 | 3.32 | 16.30 | 8.84E-08 |
| Japan | Female | 1999 | 1.34 | 6.62 | 2.97E-08 |
| Japan | Both | 1999 | 2.16 | 10.66 | 5.79E-08 |
| Japan | Male | 2000 | 3.17 | 15.61 | 8.54E-08 |
| Japan | Female | 2000 | 1.27 | 6.30 | 2.52E-08 |
| Japan | Both | 2000 | 2.06 | 10.19 | 5.24E-08 |
| Japan | Male | 2001 | 3.04 | 15.01 | 8.10E-08 |
| Japan | Female | 2001 | 1.21 | 6.03 | 2.31E-08 |
| Japan | Both | 2001 | 1.97 | 9.77 | 5.70E-08 |
| Japan | Male | 2002 | 2.91 | 14.36 | 8.61E-08 |
| Japan | Female | 2002 | 1.16 | 5.79 | 1.98E-08 |
| Japan | Both | 2002 | 1.89 | 9.38 | 5.09E-08 |
| Japan | Male | 2003 | 2.85 | 14.12 | 7.61E-08 |
| Japan | Female | 2003 | 1.11 | 5.56 | 1.60E-08 |
| Japan | Both | 2003 | 1.84 | 9.13 | 4.33E-08 |
| Japan | Male | 2004 | 2.80 | 13.83 | 8.12E-08 |
| Japan | Female | 2004 | 1.10 | 5.49 | 1.46E-08 |
| Japan | Both | 2004 | 1.81 | 8.97 | 4.36E-08 |
| Japan | Male | 2005 | 2.73 | 13.51 | 7.55E-08 |
| Japan | Female | 2005 | 1.06 | 5.27 | 1.16E-08 |
| Japan | Both | 2005 | 1.76 | 8.73 | 3.88E-08 |
| Japan | Male | 2006 | 2.64 | 13.08 | 6.69E-08 |
| Japan | Female | 2006 | 1.02 | 5.09 | 1.01E-08 |
| Japan | Both | 2006 | 1.70 | 8.48 | 3.46E-08 |
| Japan | Male | 2007 | 2.57 | 12.74 | 6.21E-08 |
| Japan | Female | 2007 | 0.98 | 4.90 | 7.99E-09 |
| Japan | Both | 2007 | 1.65 | 8.25 | 3.06E-08 |
| Japan | Male | 2008 | 2.50 | 12.39 | 5.08E-08 |
| Japan | Female | 2008 | 0.94 | 4.72 | 6.48E-09 |
| Japan | Both | 2008 | 1.60 | 7.99 | 2.54E-08 |
| Japan | Male | 2009 | 2.45 | 12.12 | 4.75E-08 |
| Japan | Female | 2009 | 0.91 | 4.60 | 5.64E-09 |
| Japan | Both | 2009 | 1.56 | 7.80 | 2.58E-08 |
| Japan | Male | 2010 | 2.39 | 11.89 | 4.43E-08 |
| Japan | Female | 2010 | 0.88 | 4.46 | 4.46E-09 |
| Japan | Both | 2010 | 1.53 | 7.63 | 2.22E-08 |
| Japan | Male | 2011 | 2.33 | 11.57 | 4.72E-08 |
| Japan | Female | 2011 | 0.87 | 4.39 | 3.68E-09 |
| Japan | Both | 2011 | 1.49 | 7.48 | 2.25E-08 |
| Japan | Male | 2012 | 2.24 | 11.07 | 4.34E-08 |
| Japan | Female | 2012 | 0.84 | 4.24 | 3.06E-09 |
| Japan | Both | 2012 | 1.44 | 7.22 | 2.07E-08 |
| Japan | Male | 2013 | 2.16 | 10.72 | 4.04E-08 |
| Japan | Female | 2013 | 0.81 | 4.10 | 2.58E-09 |
| Japan | Both | 2013 | 1.39 | 6.99 | 1.95E-08 |
| Japan | Male | 2014 | 2.08 | 10.30 | 3.78E-08 |
| Japan | Female | 2014 | 0.78 | 3.98 | 2.06E-09 |
| Japan | Both | 2014 | 1.34 | 6.76 | 1.85E-08 |
| Japan | Male | 2015 | 2.00 | 9.93 | 3.35E-08 |
| Japan | Female | 2015 | 0.74 | 3.80 | 1.91E-09 |
| Japan | Both | 2015 | 1.29 | 6.50 | 1.70E-08 |
| Japan | Male | 2016 | 1.92 | 9.58 | 3.02E-08 |
| Japan | Female | 2016 | 0.72 | 3.69 | 1.71E-09 |
| Japan | Both | 2016 | 1.25 | 6.26 | 1.60E-08 |
| Japan | Male | 2017 | 1.85 | 9.20 | 3.02E-08 |
| Japan | Female | 2017 | 0.69 | 3.53 | 1.41E-09 |
| Japan | Both | 2017 | 1.20 | 5.97 | 1.58E-08 |
| Japan | Male | 2018 | 1.78 | 8.84 | 2.46E-08 |
| Japan | Female | 2018 | 0.67 | 3.44 | 1.32E-09 |
| Japan | Both | 2018 | 1.16 | 5.79 | 1.31E-08 |
| Japan | Male | 2019 | 1.72 | 8.59 | 2.25E-08 |
| Japan | Female | 2019 | 0.65 | 3.33 | 1.29E-09 |
| Japan | Both | 2019 | 1.12 | 5.61 | 1.16E-08 |
| Japan | Male | 2020 | 1.67 | 8.38 | 1.84E-08 |
| Japan | Female | 2020 | 0.62 | 3.20 | 1.39E-09 |
| Japan | Both | 2020 | 1.08 | 5.42 | 1.01E-08 |
| Japan | Male | 2021 | 1.68 | 8.45 | 2.05E-08 |
| Japan | Female | 2021 | 0.63 | 3.22 | 1.38E-09 |
| Japan | Both | 2021 | 1.09 | 5.46 | 1.08E-08 |
| China | Male | 1990 | 5.42 | 26.71 | -2.57E-07 |
| China | Female | 1990 | 2.48 | 12.12 | -1.89E-08 |
| China | Both | 1990 | 3.85 | 18.79 | -8.80E-08 |
| China | Male | 1991 | 5.30 | 26.67 | -2.42E-07 |
| China | Female | 1991 | 2.41 | 11.96 | -7.61E-09 |
| China | Both | 1991 | 3.76 | 18.84 | -9.20E-08 |
| China | Male | 1992 | 5.20 | 25.82 | -2.32E-07 |
| China | Female | 1992 | 2.33 | 11.57 | -7.29E-09 |
| China | Both | 1992 | 3.67 | 18.01 | -1.05E-07 |
| China | Male | 1993 | 5.03 | 24.96 | -1.72E-07 |
| China | Female | 1993 | 2.30 | 11.51 | -6.25E-09 |
| China | Both | 1993 | 3.58 | 17.78 | -8.79E-08 |
| China | Male | 1994 | 4.92 | 24.74 | -1.76E-07 |
| China | Female | 1994 | 2.23 | 11.33 | 2.64E-09 |
| China | Both | 1994 | 3.49 | 17.50 | -8.09E-08 |
| China | Male | 1995 | 4.83 | 24.25 | -1.45E-07 |
| China | Female | 1995 | 2.18 | 10.95 | 9.77E-09 |
| China | Both | 1995 | 3.42 | 17.08 | -7.85E-08 |
| China | Male | 1996 | 4.71 | 23.39 | -1.15E-07 |
| China | Female | 1996 | 2.11 | 10.37 | 9.41E-09 |
| China | Both | 1996 | 3.33 | 16.44 | -7.29E-08 |
| China | Male | 1997 | 4.62 | 22.89 | -1.20E-07 |
| China | Female | 1997 | 2.04 | 9.88 | 1.64E-08 |
| China | Both | 1997 | 3.25 | 15.93 | -5.40E-08 |
| China | Male | 1998 | 4.57 | 22.61 | -1.43E-07 |
| China | Female | 1998 | 1.99 | 9.67 | 2.29E-08 |
| China | Both | 1998 | 3.20 | 15.80 | -4.71E-08 |
| China | Male | 1999 | 4.57 | 23.58 | -1.23E-07 |
| China | Female | 1999 | 1.98 | 9.72 | 8.99E-09 |
| China | Both | 1999 | 3.19 | 15.84 | -4.04E-08 |
| China | Male | 2000 | 4.60 | 22.82 | -1.33E-07 |
| China | Female | 2000 | 1.98 | 10.06 | 1.17E-08 |
| China | Both | 2000 | 3.21 | 15.68 | -4.64E-08 |
| China | Male | 2001 | 4.69 | 23.15 | -1.22E-07 |
| China | Female | 2001 | 1.97 | 9.76 | 1.35E-08 |
| China | Both | 2001 | 3.24 | 15.89 | -3.64E-08 |
| China | Male | 2002 | 4.66 | 23.19 | -1.36E-07 |
| China | Female | 2002 | 1.95 | 9.51 | 1.76E-08 |
| China | Both | 2002 | 3.21 | 15.74 | -4.25E-08 |
| China | Male | 2003 | 4.72 | 23.54 | -9.50E-08 |
| China | Female | 2003 | 1.94 | 9.65 | 1.27E-08 |
| China | Both | 2003 | 3.22 | 15.76 | -3.60E-08 |
| China | Male | 2004 | 4.77 | 23.80 | -8.81E-08 |
| China | Female | 2004 | 1.93 | 9.82 | 1.75E-08 |
| China | Both | 2004 | 3.24 | 15.85 | -3.55E-08 |
| China | Male | 2005 | 4.62 | 22.83 | -9.40E-08 |
| China | Female | 2005 | 1.83 | 9.08 | 2.27E-08 |
| China | Both | 2005 | 3.12 | 15.22 | -2.93E-08 |
| China | Male | 2006 | 4.24 | 21.02 | -8.69E-08 |
| China | Female | 2006 | 1.66 | 8.25 | 2.46E-08 |
| China | Both | 2006 | 2.85 | 14.11 | -2.34E-08 |
| China | Male | 2007 | 4.01 | 19.82 | -8.91E-08 |
| China | Female | 2007 | 1.55 | 7.69 | 2.92E-08 |
| China | Both | 2007 | 2.68 | 13.18 | -2.10E-08 |
| China | Male | 2008 | 3.94 | 19.70 | -9.48E-08 |
| China | Female | 2008 | 1.47 | 7.41 | 1.85E-08 |
| China | Both | 2008 | 2.61 | 13.04 | -1.10E-08 |
| China | Male | 2009 | 3.85 | 19.19 | -8.42E-08 |
| China | Female | 2009 | 1.43 | 7.10 | 9.66E-09 |
| China | Both | 2009 | 2.55 | 12.56 | -1.38E-08 |
| China | Male | 2010 | 3.75 | 18.94 | -8.76E-08 |
| China | Female | 2010 | 1.38 | 7.02 | 7.72E-09 |
| China | Both | 2010 | 2.48 | 12.39 | -1.60E-08 |
| China | Male | 2011 | 3.60 | 17.58 | -8.52E-08 |
| China | Female | 2011 | 1.29 | 6.37 | 1.35E-08 |
| China | Both | 2011 | 2.36 | 11.44 | -2.52E-08 |
| China | Male | 2012 | 3.48 | 17.06 | -6.62E-08 |
| China | Female | 2012 | 1.22 | 6.07 | 1.34E-08 |
| China | Both | 2012 | 2.27 | 11.28 | -2.52E-08 |
| China | Male | 2013 | 3.35 | 16.42 | -5.83E-08 |
| China | Female | 2013 | 1.16 | 5.77 | 7.65E-09 |
| China | Both | 2013 | 2.17 | 10.59 | -1.33E-08 |
| China | Male | 2014 | 3.21 | 16.11 | -5.96E-08 |
| China | Female | 2014 | 1.10 | 5.54 | 6.68E-09 |
| China | Both | 2014 | 2.08 | 10.43 | -1.97E-08 |
| China | Male | 2015 | 3.07 | 15.10 | -7.19E-08 |
| China | Female | 2015 | 1.08 | 5.56 | 7.60E-09 |
| China | Both | 2015 | 2.00 | 9.72 | -2.63E-08 |
| China | Male | 2016 | 3.04 | 15.26 | -7.26E-08 |
| China | Female | 2016 | 1.06 | 5.34 | 8.26E-09 |
| China | Both | 2016 | 1.97 | 9.80 | -2.84E-08 |
| China | Male | 2017 | 2.92 | 14.74 | -6.14E-08 |
| China | Female | 2017 | 1.05 | 5.39 | 5.80E-09 |
| China | Both | 2017 | 1.91 | 9.62 | -2.05E-08 |
| China | Male | 2018 | 2.86 | 14.80 | -5.51E-08 |
| China | Female | 2018 | 1.03 | 5.13 | 1.17E-08 |
| China | Both | 2018 | 1.88 | 9.40 | -2.28E-08 |
| China | Male | 2019 | 2.81 | 15.03 | -6.38E-08 |
| China | Female | 2019 | 1.01 | 5.17 | 7.03E-09 |
| China | Both | 2019 | 1.85 | 9.50 | -2.34E-08 |
| China | Male | 2020 | 2.76 | 13.80 | -5.80E-08 |
| China | Female | 2020 | 1.01 | 5.07 | 6.13E-09 |
| China | Both | 2020 | 1.82 | 8.72 | -1.70E-08 |
| China | Male | 2021 | 2.71 | 14.07 | -5.21E-08 |
| China | Female | 2021 | 0.99 | 5.06 | 9.71E-09 |
| China | Both | 2021 | 1.78 | 8.81 | -2.78E-08 |
| South Korea | Male | 1990 | 7.19 | 35.74 | -4.34E-08 |
| South Korea | Female | 1990 | 3.00 | 14.66 | 8.02E-09 |
| South Korea | Both | 1990 | 4.61 | 22.14 | 6.24E-08 |
| South Korea | Male | 1991 | 7.01 | 34.68 | -8.94E-09 |
| South Korea | Female | 1991 | 2.85 | 13.97 | 1.22E-08 |
| South Korea | Both | 1991 | 4.45 | 21.66 | 5.23E-08 |
| South Korea | Male | 1992 | 6.68 | 33.16 | 8.64E-09 |
| South Korea | Female | 1992 | 2.70 | 13.17 | 1.60E-08 |
| South Korea | Both | 1992 | 4.22 | 20.70 | 4.03E-08 |
| South Korea | Male | 1993 | 6.49 | 32.45 | -3.07E-09 |
| South Korea | Female | 1993 | 2.57 | 12.72 | 1.14E-08 |
| South Korea | Both | 1993 | 4.07 | 20.03 | 4.81E-08 |
| South Korea | Male | 1994 | 6.43 | 32.49 | 3.91E-09 |
| South Korea | Female | 1994 | 2.47 | 12.19 | 1.43E-08 |
| South Korea | Both | 1994 | 3.97 | 19.65 | 4.25E-08 |
| South Korea | Male | 1995 | 6.07 | 30.78 | -7.78E-09 |
| South Korea | Female | 1995 | 2.39 | 11.77 | 2.11E-08 |
| South Korea | Both | 1995 | 3.80 | 18.91 | 4.11E-08 |
| South Korea | Male | 1996 | 5.76 | 29.14 | -6.76E-09 |
| South Korea | Female | 1996 | 2.27 | 11.11 | 1.97E-08 |
| South Korea | Both | 1996 | 3.62 | 18.06 | 4.95E-08 |
| South Korea | Male | 1997 | 5.48 | 27.77 | -5.63E-09 |
| South Korea | Female | 1997 | 2.17 | 10.63 | 8.75E-09 |
| South Korea | Both | 1997 | 3.45 | 17.12 | 5.07E-08 |
| South Korea | Male | 1998 | 5.26 | 26.46 | -4.24E-09 |
| South Korea | Female | 1998 | 2.08 | 10.23 | 1.28E-08 |
| South Korea | Both | 1998 | 3.30 | 16.26 | 5.22E-08 |
| South Korea | Male | 1999 | 5.04 | 25.10 | -4.18E-08 |
| South Korea | Female | 1999 | 2.01 | 10.07 | 1.11E-08 |
| South Korea | Both | 1999 | 3.17 | 15.64 | 3.86E-08 |
| South Korea | Male | 2000 | 4.75 | 23.37 | -5.53E-08 |
| South Korea | Female | 2000 | 1.89 | 9.45 | 1.61E-08 |
| South Korea | Both | 2000 | 3.00 | 14.76 | 3.80E-08 |
| South Korea | Male | 2001 | 4.40 | 21.33 | 2.78E-09 |
| South Korea | Female | 2001 | 1.75 | 8.72 | 2.56E-09 |
| South Korea | Both | 2001 | 2.77 | 13.54 | 1.39E-08 |
| South Korea | Male | 2002 | 4.11 | 19.92 | -1.65E-08 |
| South Korea | Female | 2002 | 1.64 | 8.04 | 1.32E-09 |
| South Korea | Both | 2002 | 2.59 | 12.56 | 1.43E-08 |
| South Korea | Male | 2003 | 3.87 | 18.89 | 9.91E-09 |
| South Korea | Female | 2003 | 1.54 | 7.49 | 6.77E-09 |
| South Korea | Both | 2003 | 2.44 | 11.84 | 2.34E-08 |
| South Korea | Male | 2004 | 3.65 | 17.93 | 2.10E-09 |
| South Korea | Female | 2004 | 1.43 | 6.97 | 6.15E-09 |
| South Korea | Both | 2004 | 2.30 | 11.25 | 1.76E-08 |
| South Korea | Male | 2005 | 3.45 | 16.85 | 3.01E-08 |
| South Korea | Female | 2005 | 1.35 | 6.63 | -6.60E-10 |
| South Korea | Both | 2005 | 2.17 | 10.61 | 1.99E-08 |
| South Korea | Male | 2006 | 3.24 | 15.90 | 2.36E-08 |
| South Korea | Female | 2006 | 1.25 | 6.21 | 6.20E-09 |
| South Korea | Both | 2006 | 2.03 | 10.06 | 3.35E-08 |
| South Korea | Male | 2007 | 3.04 | 15.11 | 1.76E-08 |
| South Korea | Female | 2007 | 1.18 | 5.88 | 4.90E-09 |
| South Korea | Both | 2007 | 1.91 | 9.46 | 1.76E-08 |
| South Korea | Male | 2008 | 2.88 | 14.31 | 1.39E-08 |
| South Korea | Female | 2008 | 1.10 | 5.49 | 9.00E-11 |
| South Korea | Both | 2008 | 1.80 | 9.03 | 2.27E-08 |
| South Korea | Male | 2009 | 2.73 | 13.53 | 1.10E-08 |
| South Korea | Female | 2009 | 1.04 | 5.16 | 3.83E-09 |
| South Korea | Both | 2009 | 1.72 | 8.58 | 3.63E-08 |
| South Korea | Male | 2010 | 2.63 | 12.96 | -2.90E-10 |
| South Korea | Female | 2010 | 1.00 | 4.93 | 1.13E-08 |
| South Korea | Both | 2010 | 1.65 | 8.23 | 2.17E-08 |
| South Korea | Male | 2011 | 2.51 | 12.33 | -6.80E-09 |
| South Korea | Female | 2011 | 0.96 | 4.75 | 4.13E-09 |
| South Korea | Both | 2011 | 1.58 | 7.83 | 8.33E-09 |
| South Korea | Male | 2012 | 2.37 | 11.62 | -2.70E-10 |
| South Korea | Female | 2012 | 0.91 | 4.54 | 1.10E-10 |
| South Korea | Both | 2012 | 1.49 | 7.47 | 2.45E-08 |
| South Korea | Male | 2013 | 2.22 | 10.95 | -4.83E-09 |
| South Korea | Female | 2013 | 0.86 | 4.41 | 5.21E-09 |
| South Korea | Both | 2013 | 1.41 | 7.05 | 2.76E-08 |
| South Korea | Male | 2014 | 2.09 | 10.27 | -1.26E-08 |
| South Korea | Female | 2014 | 0.81 | 4.14 | 3.82E-09 |
| South Korea | Both | 2014 | 1.33 | 6.65 | 2.55E-08 |
| South Korea | Male | 2015 | 2.00 | 9.89 | -1.75E-08 |
| South Korea | Female | 2015 | 0.78 | 3.99 | 4.08E-09 |
| South Korea | Both | 2015 | 1.28 | 6.38 | 1.91E-08 |
| South Korea | Male | 2016 | 1.93 | 9.61 | -1.51E-08 |
| South Korea | Female | 2016 | 0.76 | 3.85 | 4.22E-09 |
| South Korea | Both | 2016 | 1.23 | 6.16 | 1.85E-08 |
| South Korea | Male | 2017 | 1.85 | 9.28 | -1.46E-08 |
| South Korea | Female | 2017 | 0.72 | 3.65 | 4.12E-09 |
| South Korea | Both | 2017 | 1.18 | 5.86 | 1.88E-08 |
| South Korea | Male | 2018 | 1.81 | 9.03 | -6.45E-09 |
| South Korea | Female | 2018 | 0.70 | 3.48 | 4.45E-09 |
| South Korea | Both | 2018 | 1.16 | 5.73 | 1.81E-08 |
| South Korea | Male | 2019 | 1.74 | 8.77 | -4.35E-09 |
| South Korea | Female | 2019 | 0.67 | 3.31 | 5.77E-09 |
| South Korea | Both | 2019 | 1.12 | 5.49 | 1.10E-08 |
| South Korea | Male | 2020 | 1.70 | 8.53 | 3.23E-09 |
| South Korea | Female | 2020 | 0.65 | 3.18 | 9.08E-09 |
| South Korea | Both | 2020 | 1.09 | 5.33 | 1.08E-08 |
| South Korea | Male | 2021 | 1.71 | 8.52 | 4.69E-09 |
| South Korea | Female | 2021 | 0.68 | 3.30 | 4.89E-09 |
| South Korea | Both | 2021 | 1.11 | 5.50 | 1.59E-08 |

Supplementary Table S2. Age-standardized DALYs rate of high sodium intake-attributed gastric cancer in China, Japan, South Korea, and the Globally from 1990 to 2021

| **location_name** | **sex_name** | **year** | **val** | **upper** | **lower** |
| --- | --- | --- | --- | --- | --- |
| China | Male | 1990 | 136.67 | 668.68 | -7.71831E-06 |
| China | Female | 1990 | 61.39 | 302.16 | -7.89E-07 |
| China | Both | 1990 | 98.40 | 478.50 | -4.31012E-06 |
| China | Male | 1991 | 133.23 | 675.08 | -7.51933E-06 |
| China | Female | 1991 | 59.64 | 295.65 | -5.29E-07 |
| China | Both | 1991 | 95.86 | 484.24 | -4.07022E-06 |
| China | Male | 1992 | 130.50 | 640.55 | -7.59164E-06 |
| China | Female | 1992 | 57.39 | 285.18 | -5.52E-07 |
| China | Both | 1992 | 93.40 | 457.40 | -4.02037E-06 |
| China | Male | 1993 | 126.01 | 626.97 | -5.76479E-06 |
| China | Female | 1993 | 56.54 | 286.82 | -5.24E-07 |
| China | Both | 1993 | 90.77 | 451.78 | -3.46919E-06 |
| China | Male | 1994 | 122.71 | 618.48 | -6.50887E-06 |
| China | Female | 1994 | 54.73 | 280.34 | -4.22E-07 |
| China | Both | 1994 | 88.24 | 446.53 | -3.44569E-06 |
| China | Male | 1995 | 120.12 | 608.65 | -5.70194E-06 |
| China | Female | 1995 | 53.39 | 265.79 | -3.32E-07 |
| China | Both | 1995 | 86.30 | 432.99 | -3.53648E-06 |
| China | Male | 1996 | 116.87 | 577.49 | -5.00436E-06 |
| China | Female | 1996 | 51.41 | 253.40 | -1.87E-07 |
| China | Both | 1996 | 83.68 | 411.39 | -2.76579E-06 |
| China | Male | 1997 | 114.00 | 563.83 | -5.19682E-06 |
| China | Female | 1997 | 49.40 | 239.30 | 1.88E-07 |
| China | Both | 1997 | 81.24 | 397.70 | -2.24247E-06 |
| China | Male | 1998 | 112.50 | 559.07 | -5.11381E-06 |
| China | Female | 1998 | 48.20 | 234.40 | 4.60E-07 |
| China | Both | 1998 | 79.88 | 393.79 | -1.86696E-06 |
| China | Male | 1999 | 111.91 | 583.13 | -4.44889E-06 |
| China | Female | 1999 | 47.78 | 232.87 | 2.41E-07 |
| China | Both | 1999 | 79.36 | 393.84 | -1.85045E-06 |
| China | Male | 2000 | 111.57 | 555.86 | -4.91507E-06 |
| China | Female | 2000 | 47.39 | 240.59 | -1.69E-08 |
| China | Both | 2000 | 78.94 | 384.33 | -1.97362E-06 |
| China | Male | 2001 | 112.93 | 561.69 | -4.68731E-06 |
| China | Female | 2001 | 46.66 | 230.35 | -1.49E-07 |
| China | Both | 2001 | 79.21 | 390.68 | -2.06341E-06 |
| China | Male | 2002 | 111.74 | 555.78 | -4.88431E-06 |
| China | Female | 2002 | 46.00 | 224.75 | 5.71E-08 |
| China | Both | 2002 | 78.25 | 381.36 | -1.90086E-06 |
| China | Male | 2003 | 111.66 | 554.50 | -4.14844E-06 |
| China | Female | 2003 | 45.25 | 226.87 | -1.77E-08 |
| China | Both | 2003 | 77.69 | 377.53 | -2.06732E-06 |
| China | Male | 2004 | 112.27 | 557.90 | -3.78666E-06 |
| China | Female | 2004 | 44.63 | 226.76 | 1.65E-07 |
| China | Both | 2004 | 77.65 | 379.29 | -1.66882E-06 |
| China | Male | 2005 | 108.08 | 534.33 | -4.16551E-06 |
| China | Female | 2005 | 42.05 | 207.94 | 3.94E-07 |
| China | Both | 2005 | 74.25 | 363.10 | -1.63116E-06 |
| China | Male | 2006 | 99.71 | 495.30 | -3.59623E-06 |
| China | Female | 2006 | 38.20 | 189.88 | 1.77E-07 |
| China | Both | 2006 | 68.20 | 335.47 | -1.35859E-06 |
| China | Male | 2007 | 94.01 | 464.04 | -3.12821E-06 |
| China | Female | 2007 | 35.76 | 175.19 | 1.52E-07 |
| China | Both | 2007 | 64.12 | 314.06 | -1.11874E-06 |
| China | Male | 2008 | 92.03 | 461.68 | -3.31746E-06 |
| China | Female | 2008 | 33.61 | 168.15 | 1.62E-07 |
| China | Both | 2008 | 62.08 | 307.41 | -9.33E-07 |
| China | Male | 2009 | 88.96 | 440.58 | -3.52323E-06 |
| China | Female | 2009 | 32.40 | 161.20 | 7.98E-08 |
| China | Both | 2009 | 59.92 | 294.37 | -1.07507E-06 |
| China | Male | 2010 | 86.28 | 434.24 | -3.53231E-06 |
| China | Female | 2010 | 31.04 | 157.68 | 6.39E-08 |
| China | Both | 2010 | 57.90 | 290.41 | -9.80E-07 |
| China | Male | 2011 | 82.90 | 403.41 | -2.87003E-06 |
| China | Female | 2011 | 28.93 | 143.23 | 8.70E-08 |
| China | Both | 2011 | 55.19 | 266.33 | -1.26067E-06 |
| China | Male | 2012 | 80.29 | 391.06 | -2.21871E-06 |
| China | Female | 2012 | 27.53 | 136.72 | 9.19E-08 |
| China | Both | 2012 | 53.18 | 261.83 | -1.16023E-06 |
| China | Male | 2013 | 76.86 | 375.94 | -1.92928E-06 |
| China | Female | 2013 | 25.98 | 129.82 | -6.64E-08 |
| China | Both | 2013 | 50.67 | 248.09 | -1.00669E-06 |
| China | Male | 2014 | 73.79 | 371.32 | -2.14124E-06 |
| China | Female | 2014 | 24.73 | 123.35 | -8.98E-08 |
| China | Both | 2014 | 48.53 | 242.79 | -1.11006E-06 |
| China | Male | 2015 | 70.35 | 349.21 | -1.64034E-06 |
| China | Female | 2015 | 24.24 | 124.48 | 3.68E-08 |
| China | Both | 2015 | 46.57 | 228.96 | -8.30E-07 |
| China | Male | 2016 | 69.54 | 354.59 | -1.65581E-06 |
| China | Female | 2016 | 23.79 | 119.63 | 1.65E-07 |
| China | Both | 2016 | 45.93 | 230.01 | -9.77E-07 |
| China | Male | 2017 | 67.09 | 341.28 | -1.70148E-06 |
| China | Female | 2017 | 23.44 | 118.69 | 5.21E-08 |
| China | Both | 2017 | 44.55 | 224.34 | -9.40E-07 |
| China | Male | 2018 | 65.76 | 341.07 | -1.93357E-06 |
| China | Female | 2018 | 22.98 | 114.61 | 1.33E-07 |
| China | Both | 2018 | 43.67 | 218.41 | -1.10686E-06 |
| China | Male | 2019 | 64.69 | 346.93 | -1.73326E-06 |
| China | Female | 2019 | 22.60 | 114.26 | 1.30E-07 |
| China | Both | 2019 | 42.94 | 221.72 | -6.86E-07 |
| China | Male | 2020 | 63.32 | 315.84 | -1.78312E-06 |
| China | Female | 2020 | 22.50 | 114.89 | 2.74E-08 |
| China | Both | 2020 | 42.21 | 204.15 | -6.09E-07 |
| China | Male | 2021 | 62.16 | 323.76 | -2.09096E-06 |
| China | Female | 2021 | 22.15 | 112.91 | 1.07E-07 |
| China | Both | 2021 | 41.46 | 208.59 | -5.01E-07 |
| Global | Male | 1990 | 62.20 | 314.71 | -1.69084E-06 |
| Global | Female | 1990 | 28.71 | 146.18 | -8.03E-08 |
| Global | Both | 1990 | 44.53 | 222.31 | -7.45E-07 |
| Global | Male | 1991 | 60.81 | 308.27 | -1.57223E-06 |
| Global | Female | 1991 | 27.99 | 141.70 | -3.98E-08 |
| Global | Both | 1991 | 43.51 | 217.97 | -7.38E-07 |
| Global | Male | 1992 | 59.76 | 297.90 | -1.67015E-06 |
| Global | Female | 1992 | 27.22 | 137.04 | -1.46E-08 |
| Global | Both | 1992 | 42.62 | 211.83 | -7.37E-07 |
| Global | Male | 1993 | 58.37 | 291.80 | -1.32346E-06 |
| Global | Female | 1993 | 26.76 | 136.75 | -3.22E-08 |
| Global | Both | 1993 | 41.73 | 207.99 | -6.67E-07 |
| Global | Male | 1994 | 57.16 | 285.77 | -1.16101E-06 |
| Global | Female | 1994 | 26.08 | 131.96 | 9.72E-09 |
| Global | Both | 1994 | 40.81 | 204.75 | -6.28E-07 |
| Global | Male | 1995 | 55.82 | 280.87 | -1.24789E-06 |
| Global | Female | 1995 | 25.41 | 127.61 | 3.87E-08 |
| Global | Both | 1995 | 39.82 | 200.54 | -5.87E-07 |
| Global | Male | 1996 | 54.33 | 269.84 | -8.83E-07 |
| Global | Female | 1996 | 24.58 | 125.37 | 1.18E-07 |
| Global | Both | 1996 | 38.68 | 192.98 | -5.13E-07 |
| Global | Male | 1997 | 52.90 | 265.88 | -8.75E-07 |
| Global | Female | 1997 | 23.77 | 118.43 | 1.56E-07 |
| Global | Both | 1997 | 37.57 | 185.81 | -3.80E-07 |
| Global | Male | 1998 | 51.99 | 259.04 | -8.20E-07 |
| Global | Female | 1998 | 23.19 | 116.38 | 1.85E-07 |
| Global | Both | 1998 | 36.83 | 183.51 | -2.83E-07 |
| Global | Male | 1999 | 51.37 | 258.91 | -7.90E-07 |
| Global | Female | 1999 | 22.85 | 113.13 | 1.88E-07 |
| Global | Both | 1999 | 36.37 | 183.15 | -2.85E-07 |
| Global | Male | 2000 | 50.56 | 251.52 | -9.16E-07 |
| Global | Female | 2000 | 22.39 | 114.49 | 1.19E-07 |
| Global | Both | 2000 | 35.73 | 179.00 | -3.72E-07 |
| Global | Male | 2001 | 50.19 | 250.07 | -8.83E-07 |
| Global | Female | 2001 | 21.87 | 108.90 | 7.88E-08 |
| Global | Both | 2001 | 35.29 | 175.84 | -3.73E-07 |
| Global | Male | 2002 | 49.29 | 241.90 | -1.01573E-06 |
| Global | Female | 2002 | 21.47 | 108.18 | 5.35E-08 |
| Global | Both | 2002 | 34.65 | 171.00 | -3.52E-07 |
| Global | Male | 2003 | 48.77 | 242.77 | -8.72E-07 |
| Global | Female | 2003 | 21.04 | 107.00 | 9.48E-08 |
| Global | Both | 2003 | 34.17 | 171.17 | -3.30E-07 |
| Global | Male | 2004 | 48.32 | 239.26 | -7.34E-07 |
| Global | Female | 2004 | 20.59 | 104.95 | 1.26E-07 |
| Global | Both | 2004 | 33.72 | 167.03 | -2.16E-07 |
| Global | Male | 2005 | 46.81 | 232.27 | -8.59E-07 |
| Global | Female | 2005 | 19.77 | 99.55 | 1.34E-07 |
| Global | Both | 2005 | 32.57 | 161.03 | -2.40E-07 |
| Global | Male | 2006 | 44.25 | 221.24 | -7.39E-07 |
| Global | Female | 2006 | 18.66 | 94.93 | 7.22E-08 |
| Global | Both | 2006 | 30.78 | 152.66 | -3.03E-07 |
| Global | Male | 2007 | 42.52 | 213.51 | -7.07E-07 |
| Global | Female | 2007 | 17.89 | 89.95 | 5.70E-08 |
| Global | Both | 2007 | 29.56 | 147.05 | -2.57E-07 |
| Global | Male | 2008 | 41.69 | 207.01 | -5.25E-07 |
| Global | Female | 2008 | 17.19 | 87.86 | 6.80E-08 |
| Global | Both | 2008 | 28.80 | 144.54 | -2.53E-07 |
| Global | Male | 2009 | 40.49 | 201.77 | -6.25E-07 |
| Global | Female | 2009 | 16.68 | 84.71 | 8.28E-08 |
| Global | Both | 2009 | 27.95 | 138.85 | -2.50E-07 |
| Global | Male | 2010 | 39.56 | 198.05 | -7.74E-07 |
| Global | Female | 2010 | 16.19 | 82.92 | 4.16E-08 |
| Global | Both | 2010 | 27.25 | 136.62 | -2.19E-07 |
| Global | Male | 2011 | 38.25 | 188.38 | -5.97E-07 |
| Global | Female | 2011 | 15.47 | 78.28 | 5.22E-08 |
| Global | Both | 2011 | 26.26 | 129.92 | -2.29E-07 |
| Global | Male | 2012 | 37.17 | 187.02 | -4.63E-07 |
| Global | Female | 2012 | 15.01 | 76.45 | 5.00E-08 |
| Global | Both | 2012 | 25.51 | 128.03 | -2.64E-07 |
| Global | Male | 2013 | 35.96 | 177.48 | -3.90E-07 |
| Global | Female | 2013 | 14.52 | 74.01 | 3.62E-08 |
| Global | Both | 2013 | 24.67 | 122.63 | -2.18E-07 |
| Global | Male | 2014 | 34.80 | 174.10 | -4.24E-07 |
| Global | Female | 2014 | 14.05 | 71.92 | 2.97E-08 |
| Global | Both | 2014 | 23.87 | 119.32 | -2.33E-07 |
| Global | Male | 2015 | 33.60 | 165.99 | -2.91E-07 |
| Global | Female | 2015 | 13.84 | 71.22 | 4.99E-08 |
| Global | Both | 2015 | 23.19 | 115.39 | -1.64E-07 |
| Global | Male | 2016 | 33.12 | 166.40 | -2.85E-07 |
| Global | Female | 2016 | 13.63 | 70.57 | 8.38E-08 |
| Global | Both | 2016 | 22.85 | 112.89 | -1.73E-07 |
| Global | Male | 2017 | 32.13 | 161.41 | -3.26E-07 |
| Global | Female | 2017 | 13.38 | 68.72 | 6.08E-08 |
| Global | Both | 2017 | 22.25 | 111.92 | -1.76E-07 |
| Global | Male | 2018 | 31.55 | 159.75 | -4.47E-07 |
| Global | Female | 2018 | 13.19 | 66.93 | 7.33E-08 |
| Global | Both | 2018 | 21.89 | 109.93 | -1.90E-07 |
| Global | Male | 2019 | 31.03 | 157.02 | -3.79E-07 |
| Global | Female | 2019 | 12.95 | 66.78 | 6.84E-08 |
| Global | Both | 2019 | 21.52 | 108.25 | -1.02E-07 |
| Global | Male | 2020 | 30.37 | 149.54 | -3.85E-07 |
| Global | Female | 2020 | 12.74 | 64.87 | 6.19E-08 |
| Global | Both | 2020 | 21.09 | 104.21 | -5.25E-08 |
| Global | Male | 2021 | 29.90 | 146.65 | -3.81E-07 |
| Global | Female | 2021 | 12.61 | 64.62 | 8.49E-08 |
| Global | Both | 2021 | 20.78 | 102.38 | -4.68E-08 |
| Japan | Male | 1990 | 93.97 | 463.10 | 2.49932E-06 |
| Japan | Female | 1990 | 43.97 | 217.15 | 1.32424E-06 |
| Japan | Both | 1990 | 65.73 | 322.56 | 2.22477E-06 |
| Japan | Male | 1991 | 91.50 | 449.48 | 2.5123E-06 |
| Japan | Female | 1991 | 42.62 | 209.64 | 1.43368E-06 |
| Japan | Both | 1991 | 63.88 | 312.63 | 1.99766E-06 |
| Japan | Male | 1992 | 88.89 | 436.93 | 2.23488E-06 |
| Japan | Female | 1992 | 41.00 | 201.95 | 1.35016E-06 |
| Japan | Both | 1992 | 61.84 | 303.53 | 1.80668E-06 |
| Japan | Male | 1993 | 85.70 | 420.10 | 2.06813E-06 |
| Japan | Female | 1993 | 39.15 | 192.60 | 1.2538E-06 |
| Japan | Both | 1993 | 59.45 | 291.54 | 1.70125E-06 |
| Japan | Male | 1994 | 83.32 | 406.94 | 1.97089E-06 |
| Japan | Female | 1994 | 37.36 | 184.71 | 1.15809E-06 |
| Japan | Both | 1994 | 57.46 | 281.21 | 1.5714E-06 |
| Japan | Male | 1995 | 80.69 | 395.83 | 1.96935E-06 |
| Japan | Female | 1995 | 36.16 | 178.55 | 9.37E-07 |
| Japan | Both | 1995 | 55.61 | 273.21 | 1.51024E-06 |
| Japan | Male | 1996 | 78.08 | 381.18 | 1.94309E-06 |
| Japan | Female | 1996 | 34.68 | 171.19 | 8.96E-07 |
| Japan | Both | 1996 | 53.67 | 262.99 | 1.41326E-06 |
| Japan | Male | 1997 | 74.75 | 365.54 | 1.83334E-06 |
| Japan | Female | 1997 | 33.04 | 163.58 | 8.85E-07 |
| Japan | Both | 1997 | 51.30 | 250.64 | 1.39133E-06 |
| Japan | Male | 1998 | 73.72 | 360.09 | 1.80935E-06 |
| Japan | Female | 1998 | 32.10 | 158.81 | 8.39E-07 |
| Japan | Both | 1998 | 50.37 | 246.52 | 1.46637E-06 |
| Japan | Male | 1999 | 71.64 | 351.65 | 2.03803E-06 |
| Japan | Female | 1999 | 31.05 | 153.96 | 7.84E-07 |
| Japan | Both | 1999 | 48.91 | 239.68 | 1.46782E-06 |
| Japan | Male | 2000 | 68.23 | 335.17 | 1.80011E-06 |
| Japan | Female | 2000 | 29.47 | 146.38 | 6.71E-07 |
| Japan | Both | 2000 | 46.55 | 228.72 | 1.31082E-06 |
| Japan | Male | 2001 | 65.23 | 320.02 | 2.09038E-06 |
| Japan | Female | 2001 | 28.00 | 139.57 | 6.09E-07 |
| Japan | Both | 2001 | 44.45 | 218.88 | 1.31215E-06 |
| Japan | Male | 2002 | 62.07 | 303.33 | 2.05281E-06 |
| Japan | Female | 2002 | 26.55 | 132.25 | 5.46E-07 |
| Japan | Both | 2002 | 42.26 | 208.09 | 1.31023E-06 |
| Japan | Male | 2003 | 60.56 | 297.72 | 1.82371E-06 |
| Japan | Female | 2003 | 25.33 | 126.58 | 4.41E-07 |
| Japan | Both | 2003 | 40.97 | 202.69 | 1.11988E-06 |
| Japan | Male | 2004 | 59.13 | 290.00 | 1.72326E-06 |
| Japan | Female | 2004 | 24.94 | 124.78 | 3.89E-07 |
| Japan | Both | 2004 | 40.13 | 198.56 | 1.06501E-06 |
| Japan | Male | 2005 | 57.55 | 283.18 | 1.71721E-06 |
| Japan | Female | 2005 | 23.79 | 119.48 | 3.12E-07 |
| Japan | Both | 2005 | 38.86 | 192.72 | 9.28E-07 |
| Japan | Male | 2006 | 55.80 | 273.79 | 1.54178E-06 |
| Japan | Female | 2006 | 22.86 | 115.02 | 2.61E-07 |
| Japan | Both | 2006 | 37.62 | 186.13 | 8.03E-07 |
| Japan | Male | 2007 | 54.00 | 264.83 | 1.37132E-06 |
| Japan | Female | 2007 | 21.86 | 110.10 | 2.21E-07 |
| Japan | Both | 2007 | 36.28 | 178.87 | 7.48E-07 |
| Japan | Male | 2008 | 52.13 | 256.25 | 1.04407E-06 |
| Japan | Female | 2008 | 20.90 | 105.00 | 1.78E-07 |
| Japan | Both | 2008 | 34.93 | 172.54 | 5.85E-07 |
| Japan | Male | 2009 | 50.82 | 249.73 | 1.11772E-06 |
| Japan | Female | 2009 | 20.08 | 101.34 | 1.44E-07 |
| Japan | Both | 2009 | 33.93 | 167.95 | 5.83E-07 |
| Japan | Male | 2010 | 49.45 | 244.13 | 1.0089E-06 |
| Japan | Female | 2010 | 19.29 | 97.56 | 1.11E-07 |
| Japan | Both | 2010 | 32.93 | 163.78 | 5.42E-07 |
| Japan | Male | 2011 | 48.11 | 237.92 | 9.70E-07 |
| Japan | Female | 2011 | 18.94 | 95.33 | 9.26E-08 |
| Japan | Both | 2011 | 32.16 | 159.91 | 4.92E-07 |
| Japan | Male | 2012 | 46.05 | 227.33 | 9.33E-07 |
| Japan | Female | 2012 | 18.25 | 91.66 | 7.84E-08 |
| Japan | Both | 2012 | 30.88 | 153.65 | 4.70E-07 |
| Japan | Male | 2013 | 44.27 | 218.81 | 9.01E-07 |
| Japan | Female | 2013 | 17.56 | 88.63 | 6.65E-08 |
| Japan | Both | 2013 | 29.71 | 148.13 | 4.57E-07 |
| Japan | Male | 2014 | 42.24 | 209.04 | 8.45E-07 |
| Japan | Female | 2014 | 16.99 | 85.41 | 5.47E-08 |
| Japan | Both | 2014 | 28.47 | 141.92 | 4.25E-07 |
| Japan | Male | 2015 | 40.43 | 200.09 | 7.85E-07 |
| Japan | Female | 2015 | 15.93 | 80.34 | 5.01E-08 |
| Japan | Both | 2015 | 27.10 | 135.06 | 4.07E-07 |
| Japan | Male | 2016 | 38.73 | 192.51 | 7.37E-07 |
| Japan | Female | 2016 | 15.33 | 77.13 | 4.60E-08 |
| Japan | Both | 2016 | 26.02 | 129.86 | 3.83E-07 |
| Japan | Male | 2017 | 37.02 | 183.71 | 6.99E-07 |
| Japan | Female | 2017 | 14.47 | 72.50 | 4.08E-08 |
| Japan | Both | 2017 | 24.80 | 124.05 | 3.54E-07 |
| Japan | Male | 2018 | 35.40 | 176.35 | 6.11E-07 |
| Japan | Female | 2018 | 14.00 | 70.52 | 3.88E-08 |
| Japan | Both | 2018 | 23.81 | 119.01 | 3.16E-07 |
| Japan | Male | 2019 | 33.95 | 169.28 | 4.99E-07 |
| Japan | Female | 2019 | 13.56 | 68.58 | 3.13E-08 |
| Japan | Both | 2019 | 22.91 | 114.75 | 2.82E-07 |
| Japan | Male | 2020 | 33.11 | 165.27 | 4.44E-07 |
| Japan | Female | 2020 | 13.08 | 66.16 | 3.77E-08 |
| Japan | Both | 2020 | 22.28 | 111.62 | 2.29E-07 |
| Japan | Male | 2021 | 33.08 | 164.98 | 3.92E-07 |
| Japan | Female | 2021 | 13.04 | 66.32 | 3.31E-08 |
| Japan | Both | 2021 | 22.26 | 111.56 | 2.09E-07 |
| South Korea | Male | 1990 | 176.68 | 876.88 | -7.85E-07 |
| South Korea | Female | 1990 | 77.39 | 378.65 | 3.42E-07 |
| South Korea | Both | 1990 | 118.73 | 580.64 | 4.54E-07 |
| South Korea | Male | 1991 | 170.87 | 850.41 | -1.2437E-06 |
| South Korea | Female | 1991 | 72.63 | 358.42 | 4.74E-07 |
| South Korea | Both | 1991 | 113.61 | 559.20 | 2.49E-07 |
| South Korea | Male | 1992 | 160.93 | 806.55 | -1.0062E-06 |
| South Korea | Female | 1992 | 67.81 | 333.37 | 3.69E-07 |
| South Korea | Both | 1992 | 106.64 | 526.88 | 8.84E-08 |
| South Korea | Male | 1993 | 154.77 | 774.89 | -4.84E-07 |
| South Korea | Female | 1993 | 63.67 | 318.08 | 3.54E-07 |
| South Korea | Both | 1993 | 101.66 | 502.07 | 6.25E-07 |
| South Korea | Male | 1994 | 151.79 | 766.63 | -5.79E-07 |
| South Korea | Female | 1994 | 60.07 | 299.97 | 5.48E-07 |
| South Korea | Both | 1994 | 98.34 | 485.96 | 9.85E-07 |
| South Korea | Male | 1995 | 142.89 | 729.93 | -3.92E-07 |
| South Korea | Female | 1995 | 57.18 | 286.52 | 6.42E-07 |
| South Korea | Both | 1995 | 93.12 | 464.27 | 1.05816E-06 |
| South Korea | Male | 1996 | 135.46 | 681.37 | 1.52E-08 |
| South Korea | Female | 1996 | 54.07 | 270.99 | 7.69E-07 |
| South Korea | Both | 1996 | 88.34 | 442.18 | 1.19221E-06 |
| South Korea | Male | 1997 | 127.08 | 640.06 | 4.03E-07 |
| South Korea | Female | 1997 | 51.22 | 257.26 | 3.76E-07 |
| South Korea | Both | 1997 | 83.11 | 413.90 | 9.59E-07 |
| South Korea | Male | 1998 | 120.53 | 600.89 | 4.83E-07 |
| South Korea | Female | 1998 | 48.30 | 240.47 | 4.16E-07 |
| South Korea | Both | 1998 | 78.67 | 388.92 | 9.02E-07 |
| South Korea | Male | 1999 | 114.12 | 560.54 | -6.61E-07 |
| South Korea | Female | 1999 | 46.11 | 231.62 | 4.21E-07 |
| South Korea | Both | 1999 | 74.71 | 369.74 | 6.90E-07 |
| South Korea | Male | 2000 | 107.28 | 526.90 | -1.50E-07 |
| South Korea | Female | 2000 | 43.45 | 217.52 | 3.00E-07 |
| South Korea | Both | 2000 | 70.34 | 345.20 | 7.89E-07 |
| South Korea | Male | 2001 | 99.00 | 482.32 | 5.42E-07 |
| South Korea | Female | 2001 | 40.19 | 197.94 | 1.94E-07 |
| South Korea | Both | 2001 | 65.01 | 316.26 | 7.79E-07 |
| South Korea | Male | 2002 | 92.07 | 444.96 | 1.00328E-06 |
| South Korea | Female | 2002 | 37.67 | 186.27 | 1.56E-07 |
| South Korea | Both | 2002 | 60.67 | 293.32 | 6.80E-07 |
| South Korea | Male | 2003 | 86.63 | 420.26 | 1.64476E-06 |
| South Korea | Female | 2003 | 35.58 | 173.55 | 1.41E-07 |
| South Korea | Both | 2003 | 57.22 | 275.91 | 7.77E-07 |
| South Korea | Male | 2004 | 81.63 | 398.02 | 1.04363E-06 |
| South Korea | Female | 2004 | 33.25 | 161.19 | 1.61E-07 |
| South Korea | Both | 2004 | 53.82 | 260.78 | 9.15E-07 |
| South Korea | Male | 2005 | 76.45 | 370.87 | 0.000001238 |
| South Korea | Female | 2005 | 31.40 | 151.62 | 1.00E-07 |
| South Korea | Both | 2005 | 50.54 | 244.71 | 8.85E-07 |
| South Korea | Male | 2006 | 71.71 | 349.81 | 1.14366E-06 |
| South Korea | Female | 2006 | 29.20 | 143.22 | 1.62E-07 |
| South Korea | Both | 2006 | 47.31 | 230.97 | 1.09637E-06 |
| South Korea | Male | 2007 | 67.30 | 333.25 | 7.64E-07 |
| South Korea | Female | 2007 | 27.73 | 136.11 | 7.31E-08 |
| South Korea | Both | 2007 | 44.61 | 218.82 | 1.00017E-06 |
| South Korea | Male | 2008 | 63.99 | 317.44 | 3.76E-07 |
| South Korea | Female | 2008 | 26.32 | 131.54 | 6.39E-08 |
| South Korea | Both | 2008 | 42.45 | 209.77 | 8.67E-07 |
| South Korea | Male | 2009 | 61.07 | 304.38 | 9.02E-07 |
| South Korea | Female | 2009 | 25.16 | 125.01 | 6.42E-08 |
| South Korea | Both | 2009 | 40.62 | 201.92 | 9.12E-07 |
| South Korea | Male | 2010 | 58.53 | 290.87 | 4.48E-07 |
| South Korea | Female | 2010 | 23.96 | 118.84 | 6.18E-08 |
| South Korea | Both | 2010 | 38.91 | 193.05 | 5.17E-07 |
| South Korea | Male | 2011 | 55.29 | 274.69 | 3.50E-07 |
| South Korea | Female | 2011 | 22.57 | 111.41 | 7.36E-08 |
| South Korea | Both | 2011 | 36.75 | 180.98 | 5.78E-07 |
| South Korea | Male | 2012 | 51.77 | 256.00 | 5.72E-07 |
| South Korea | Female | 2012 | 21.19 | 105.28 | 2.70E-08 |
| South Korea | Both | 2012 | 34.49 | 169.51 | 7.77E-07 |
| South Korea | Male | 2013 | 48.19 | 237.22 | 4.27E-07 |
| South Korea | Female | 2013 | 19.96 | 100.79 | 1.71E-07 |
| South Korea | Both | 2013 | 32.28 | 158.82 | 8.15E-07 |
| South Korea | Male | 2014 | 45.06 | 220.32 | 3.06E-07 |
| South Korea | Female | 2014 | 18.73 | 95.13 | 1.43E-07 |
| South Korea | Both | 2014 | 30.26 | 148.41 | 6.56E-07 |
| South Korea | Male | 2015 | 42.59 | 207.65 | 7.88E-08 |
| South Korea | Female | 2015 | 17.81 | 90.26 | 1.64E-07 |
| South Korea | Both | 2015 | 28.67 | 140.90 | 5.65E-07 |
| South Korea | Male | 2016 | 40.59 | 200.36 | 4.27E-08 |
| South Korea | Female | 2016 | 17.02 | 86.32 | 1.55E-07 |
| South Korea | Both | 2016 | 27.38 | 134.98 | 5.14E-07 |
| South Korea | Male | 2017 | 38.59 | 193.45 | 1.09E-08 |
| South Korea | Female | 2017 | 16.04 | 79.49 | 1.28E-07 |
| South Korea | Both | 2017 | 25.99 | 127.46 | 5.16E-07 |
| South Korea | Male | 2018 | 37.53 | 188.61 | -9.42E-09 |
| South Korea | Female | 2018 | 15.58 | 76.24 | 9.38E-08 |
| South Korea | Both | 2018 | 25.30 | 123.78 | 5.56E-07 |
| South Korea | Male | 2019 | 36.19 | 181.96 | -9.08E-08 |
| South Korea | Female | 2019 | 15.07 | 73.82 | 1.19E-07 |
| South Korea | Both | 2019 | 24.46 | 119.11 | 4.07E-07 |
| South Korea | Male | 2020 | 35.01 | 175.03 | -1.09E-07 |
| South Korea | Female | 2020 | 14.74 | 72.52 | 9.98E-08 |
| South Korea | Both | 2020 | 23.73 | 116.16 | 4.09E-07 |
| South Korea | Male | 2021 | 35.20 | 176.51 | 2.87E-08 |
| South Korea | Female | 2021 | 15.09 | 74.72 | 7.68E-08 |
| South Korea | Both | 2021 | 24.08 | 117.95 | 5.14E-07 |

Supplementary Table S3. Age-Specific DALYs Rates of High Sodium Intake-Attributed Gastric Cancer in Globally ,2021, by Sex

| **sex_name** | **age_name** | **val** | **upper** | **lower** |
| --- | --- | --- | --- | --- |
| Male | 25-29 years | 2.357195921 | 11.89222943 | -5.89E-08 |
| Female | 25-29 years | 2.440547466 | 12.7015577 | -3.48E-08 |
| Male | 30-34 years | 6.269781007 | 31.2339338 | -1.71E-07 |
| Female | 30-34 years | 4.75204449 | 24.42446793 | -6.18E-08 |
| Male | 35-39 years | 11.29974329 | 55.82542882 | -3.59E-07 |
| Female | 35-39 years | 7.407545311 | 38.37675681 | -1.69E-07 |
| Male | 40-44 years | 20.58482022 | 100.0238007 | -1.01032E-06 |
| Female | 40-44 years | 10.98881483 | 56.17205847 | -1.98E-07 |
| Male | 45-49 years | 33.77116055 | 166.919257 | -1.47295E-06 |
| Female | 45-49 years | 15.27800328 | 78.80463974 | -2.97E-07 |
| Male | 50-54 years | 58.52021607 | 287.9985714 | -2.60975E-06 |
| Female | 50-54 years | 22.68288865 | 116.6798055 | -5.51E-07 |
| Male | 55-59 years | 85.47304981 | 423.426023 | -3.82374E-06 |
| Female | 55-59 years | 31.65453809 | 161.8510094 | -5.65E-07 |
| Male | 60-64 years | 109.5205457 | 546.7710266 | -3.39191E-06 |
| Female | 60-64 years | 40.43474317 | 205.0935116 | -6.14E-07 |
| Male | 65-69 years | 144.876232 | 711.3899914 | -6.18141E-06 |
| Female | 65-69 years | 54.98410399 | 281.7030817 | -8.46E-07 |
| Male | 70-74 years | 172.0351377 | 847.9177548 | -4.83198E-06 |
| Female | 70-74 years | 66.48551919 | 339.2349122 | -6.87E-07 |
| Male | 75-79 years | 177.7021738 | 874.397422 | -1.8203E-06 |
| Female | 75-79 years | 75.18901626 | 384.9292404 | -2.25E-07 |
| Male | 80-84 years | 177.4267068 | 875.5190106 | 7.05E-08 |
| Female | 80-84 years | 80.01786314 | 415.2385782 | 4.99E-08 |
| Male | 85-89 years | 191.0654978 | 955.5517658 | -3.25E-07 |
| Female | 85-89 years | 84.04987496 | 438.8371757 | 1.41E-07 |
| Male | 90-94 years | 187.2623265 | 942.9846579 | 7.24E-07 |
| Female | 90-94 years | 99.68438246 | 525.8623415 | 1.86E-08 |
| Male | 95+ years | 148.4320986 | 755.6244041 | 1.57E-07 |
| Female | 95+ years | 116.1035272 | 613.6076468 | 1.26E-07 |

Supplementary Table S4. Age-Specific DALYs Rates of High Sodium Intake-Attributed Gastric Cancer in China ,2021, by Sex

| **sex_name** | **age_name** | **val** | **upper** | **lower** |
| --- | --- | --- | --- | --- |
| Male | 25-29 years | 5.755566155 | 28.68959514 | -4.53E-07 |
| Female | 25-29 years | 3.850981311 | 19.31538181 | -2.64E-07 |
| Male | 30-34 years | 14.80031026 | 74.57639587 | -9.58E-07 |
| Female | 30-34 years | 7.185096713 | 36.05157833 | -3.15E-07 |
| Male | 35-39 years | 27.31962678 | 135.5055901 | -2.23981E-06 |
| Female | 35-39 years | 11.31845768 | 56.03658989 | -9.18E-07 |
| Male | 40-44 years | 49.59572782 | 248.7580708 | -5.80914E-06 |
| Female | 40-44 years | 17.48902727 | 89.03217662 | -1.15611E-06 |
| Male | 45-49 years | 71.14974304 | 374.7795953 | -7.82836E-06 |
| Female | 45-49 years | 21.85386399 | 109.5904861 | -1.36654E-06 |
| Male | 50-54 years | 116.9472929 | 616.2496735 | -1.0521E-05 |
| Female | 50-54 years | 34.97007408 | 177.1114024 | -2.14986E-06 |
| Male | 55-59 years | 165.6988194 | 876.4915235 | -1.42141E-05 |
| Female | 55-59 years | 50.49434622 | 253.7419431 | -2.56793E-06 |
| Male | 60-64 years | 225.9451417 | 1181.135914 | -1.84009E-05 |
| Female | 60-64 years | 73.36388299 | 365.0772083 | -3.55891E-06 |
| Male | 65-69 years | 279.0556062 | 1426.646321 | -2.2428E-05 |
| Female | 65-69 years | 99.77475861 | 500.4458741 | -3.86585E-06 |
| Male | 70-74 years | 357.1773338 | 1865.502628 | -1.80627E-05 |
| Female | 70-74 years | 134.9373904 | 676.8309842 | -3.04395E-06 |
| Male | 75-79 years | 378.7333478 | 1926.98196 | -1.10285E-05 |
| Female | 75-79 years | 152.6835083 | 755.7037525 | -1.93672E-06 |
| Male | 80-84 years | 367.0090066 | 1824.5987 | -1.04428E-06 |
| Female | 80-84 years | 159.6273302 | 818.8429789 | -5.74E-07 |
| Male | 85-89 years | 434.9460137 | 2152.834165 | -5.04868E-06 |
| Female | 85-89 years | 150.2966393 | 763.6555262 | 4.04E-08 |
| Male | 90-94 years | 461.9480384 | 2315.044264 | 4.04E-07 |
| Female | 90-94 years | 159.5002289 | 816.9202954 | -6.51E-07 |
| Male | 95+ years | 346.2969568 | 1727.035849 | -1.8504E-06 |
| Female | 95+ years | 176.3077509 | 892.3305173 | -1.98E-07 |

Supplementary Table S5. Age-Specific DALYs Rates of High Sodium Intake-Attributed Gastric Cancer in Japan ,2021, by Sex

| **sex_name** | **age_name** | **val** | **upper** | **lower** |
| --- | --- | --- | --- | --- |
| Male | 25-29 years | 2.381874946 | 11.93190767 | -4.30E-10 |
| Female | 25-29 years | 2.799224068 | 14.08172968 | 1.02E-09 |
| Male | 30-34 years | 4.324219798 | 21.66385764 | -5.62E-09 |
| Female | 30-34 years | 5.12673628 | 25.7623942 | -2.76E-09 |
| Male | 35-39 years | 8.003346873 | 39.46531133 | 2.20E-10 |
| Female | 35-39 years | 8.077846971 | 40.83795716 | 5.79E-09 |
| Male | 40-44 years | 12.10818011 | 60.3500124 | -2.16E-08 |
| Female | 40-44 years | 12.01620997 | 61.06084908 | 1.05E-09 |
| Male | 45-49 years | 21.1010885 | 104.5407718 | 3.29E-08 |
| Female | 45-49 years | 15.10131285 | 76.39115997 | 1.89E-08 |
| Male | 50-54 years | 35.72581727 | 178.2489239 | -8.06E-09 |
| Female | 50-54 years | 19.77071876 | 99.35650812 | 1.02E-08 |
| Male | 55-59 years | 66.5016567 | 330.4262171 | 3.61E-07 |
| Female | 55-59 years | 26.79591406 | 133.7725007 | 8.17E-09 |
| Male | 60-64 years | 111.6969488 | 548.8170803 | -3.80E-07 |
| Female | 60-64 years | 35.83111423 | 178.507115 | -2.36E-08 |
| Male | 65-69 years | 169.2372261 | 835.792935 | 3.13929E-06 |
| Female | 65-69 years | 49.66948875 | 248.423059 | -1.74E-08 |
| Male | 70-74 years | 221.0071715 | 1092.856274 | 3.51493E-06 |
| Female | 70-74 years | 60.23876379 | 303.0833259 | 8.42E-09 |
| Male | 75-79 years | 267.9930051 | 1337.880513 | 1.47366E-06 |
| Female | 75-79 years | 77.98303481 | 401.9557777 | 1.82E-07 |
| Male | 80-84 years | 308.3771621 | 1565.389551 | 4.43E-07 |
| Female | 80-84 years | 98.98195084 | 515.7327653 | 7.76E-08 |
| Male | 85-89 years | 379.3631254 | 1889.748257 | 3.26E-07 |
| Female | 85-89 years | 149.5198424 | 800.001362 | 4.46E-08 |
| Male | 90-94 years | 447.1469406 | 2228.91511 | -1.4351E-06 |
| Female | 90-94 years | 231.7855398 | 1203.977281 | -3.16E-07 |
| Male | 95+ years | 324.7652961 | 1651.822728 | -1.65899E-06 |
| Female | 95+ years | 273.8128087 | 1422.092758 | -1.08123E-06 |

Supplementary Table S6. Age-Specific DALYs Rates of High Sodium Intake-Attributed Gastric Cancer in South Korea ,2021, by Sex

| **sex_name** | **age_name** | **val** | **upper** | **lower** |
| --- | --- | --- | --- | --- |
| Male | 25-29 years | 2.723413795 | 14.79794949 | -4.89E-08 |
| Female | 25-29 years | 4.083707902 | 21.04768537 | -8.49E-09 |
| Male | 30-34 years | 5.881955419 | 30.57315823 | -9.79E-08 |
| Female | 30-34 years | 8.992389937 | 48.04464239 | -2.77E-08 |
| Male | 35-39 years | 11.36055157 | 61.45562164 | -2.66E-07 |
| Female | 35-39 years | 17.27283524 | 91.1373945 | -9.48E-08 |
| Male | 40-44 years | 19.82443729 | 105.9467214 | -3.55E-07 |
| Female | 40-44 years | 21.28171574 | 103.3551 | -2.23E-07 |
| Male | 45-49 years | 34.83780122 | 175.136221 | -7.38E-07 |
| Female | 45-49 years | 25.02750568 | 122.846706 | -2.56E-07 |
| Male | 50-54 years | 57.66185611 | 300.2600661 | -1.38173E-06 |
| Female | 50-54 years | 25.79729494 | 134.9270801 | -2.43E-07 |
| Male | 55-59 years | 86.50739491 | 442.2752175 | -1.71085E-06 |
| Female | 55-59 years | 27.1166437 | 145.6003078 | -3.65E-07 |
| Male | 60-64 years | 112.313997 | 571.8880374 | -3.32713E-06 |
| Female | 60-64 years | 29.67269077 | 158.2063621 | -6.34E-07 |
| Male | 65-69 years | 141.2733876 | 712.5675941 | -3.16713E-06 |
| Female | 65-69 years | 34.56495281 | 181.4433745 | -5.33E-07 |
| Male | 70-74 years | 177.730897 | 906.7852088 | -3.74933E-06 |
| Female | 70-74 years | 48.2372868 | 250.992733 | -7.71E-07 |
| Male | 75-79 years | 262.240905 | 1397.246677 | -5.73966E-06 |
| Female | 75-79 years | 87.52876872 | 444.1083359 | -1.47856E-06 |
| Male | 80-84 years | 350.3942514 | 1784.234608 | -1.12156E-05 |
| Female | 80-84 years | 132.5848908 | 696.5654237 | -2.84343E-06 |
| Male | 85-89 years | 378.8554695 | 1929.29232 | -7.37871E-06 |
| Female | 85-89 years | 169.5167715 | 872.2390517 | -2.13178E-06 |
| Male | 90-94 years | 414.0722564 | 2088.467165 | -6.47003E-06 |
| Female | 90-94 years | 212.1196871 | 1106.218637 | -1.89193E-06 |
| Male | 95+ years | 427.6765338 | 2258.811001 | -8.16379E-06 |
| Female | 95+ years | 246.3582247 | 1276.461264 | -3.50427E-06 |
